# Whole-genome sequence of synthetically derived *Brassica napus* inbred cultivar Da-Ae

**DOI:** 10.1093/g3journal/jkad026

**Published:** 2023-02-01

**Authors:** John T Davis, Ruijuan Li, Seungmo Kim, Richard Michelmore, Shinje Kim, Julin N Maloof

**Affiliations:** Department of Plant Biology, University of California, Davis, Davis, CA 95616, USA; Department of Plant Biology, University of California, Davis, Davis, CA 95616, USA; Present address: Inari Agriculture, Cambridge, MA 02139, USA; FnP Co., Ltd., Jeungpyeong-gun, Chungbuk-do 27903, South Korea; Genome Center and Department of Plant Sciences, University of California, Davis, Davis, CA 95616, USA; FnP Co., Ltd., Jeungpyeong-gun, Chungbuk-do 27903, South Korea; Department of Plant Biology, University of California, Davis, Davis, CA 95616, USA

**Keywords:** Illumina, Dovetail, scaffolds, allotetraploid, subgenome

## Abstract

*Brassica napus*, a globally important oilseed crop, is an allotetraploid hybrid species with two subgenomes originating from *Brassica rapa* and *Brassica oleracea.* The presence of two highly similar subgenomes has made the assembly of a complete draft genome challenging and has also resulted in natural homoeologous exchanges between the genomes, resulting in variations in gene copy number, which further complicates assigning sequences to correct chromosomes. Despite these challenges, high-quality draft genomes of this species have been released. Using third generation sequencing and assembly technologies, we generated a new genome assembly for the synthetic *B. napus* cultivar Da-Ae. Through the use of long reads, linked-reads, and Hi-C proximity data, we assembled a new draft genome that provides a high-quality reference genome of a synthetic *B. napus*. In addition, we identified potential hotspots of homoeologous exchange between subgenomes within Da-Ae, based on their presence in other independently derived lines. The occurrence of these hotspots may provide insight into the genetic rearrangements required for *B. napus* to be viable following the hybridization of *B. rapa* and *B. oleracea*.

## Introduction

*Brassica napus*, commonly known as rapeseed, is the second most widely cultivated oilseed crop in the world (USDA). Historically, rapeseed oil was used primarily in the production of lubricants due to its high erucic acid content. In the late 1970s, new, edible, low erucic acid cultivars were created, enabling rapeseed oil to become a major component of most commercial vegetable oil products (Oplinger *et al.* 1989). The demand for rapeseed oil has caused global production to more than triple in the last few decades, with China and Canada being the world's largest producers (PSD Online 2020). Numerous attempts are being made to understand the biology of *B. napus* with the goal of increasing production to keep up with demand.

The genetics of *B. napus* is challenging to untangle due to its genomic complexity. *Brassica napus* is an outcrossing species that originated from the hybridization of 2 different diploid parents, *Brassica rapa* and *Brassica oleracea* (Nagaharu 1935). Both *B. rapa* and *B. oleracea* are widely cultivated as human food crops such as cabbage, bok choy, and broccoli. It is believed that *B. napus* first appeared ∼7,500 years ago when *B. rapa* hybridized with *B. oleracea* and underwent a chromosome doubling event, resulting in an allotetraploid (Chalhoub *et al.* 2014). *Brassica napus* (AACC) contains the diploid genomes of both *B. rapa* (AA) and *B. oleracea* (CC). While polyploidy has been hypothesized to provide plants with advantages, such as favorability in domestication (Bertioli *et al.* 2019), it also has genetic consequences that can cause several analytical challenges. In the case of *B. napus*, the A and C subgenomes are so similar that there can be homoeologous exchange of genetic information between the 2 subgenomes. Such exchanges range in size from a few base pairs (gene conversion) to larger chromosomal regions (Chalhoub *et al.* 2014). The rate and specifics of homoeologous exchange varies between *B. napus* populations. Homoeologous exchange and aneuploidy occur more often in populations that have a newly synthesized *B. napus* as a parent (Udall *et al.* 2005; Xiong *et al.* 2011, 2021; Higgins *et al.* 2018; Ferreira de Carvalho *et al.* 2021) and loci affecting the homoeologous exchange rate have been identified (Higgins *et al.* 2021). Homoeologous exchange is thought to be a driving factor in the large amount of diversity found within *B. napus* (Gaeta *et al.* 2007; Stein *et al.* 2017; Higgins *et al.* 2018; Hurgobin *et al.* 2018; Lloyd *et al.* 2018; Raman *et al.* 2022). Consequently, it is important to have genome assemblies from multiple different *B. napus* varieties as an aid to building a pan-genome for this species.

In 2014, a genomic reference assembly for *B. napus* was released to the public (Chalhoub *et al.* 2014). This assembly, herein referred to as Darmor-bzh, was generated using short read sequencing data. Due to challenges associated with assembling and scaffolding short reads and the high similarity between the 2 subgenomes, a significant portion of the genome could not be confidently anchored in the assembly and was left unscaffolded (Table 1). Since the release of the Darmor-bzh assembly, new sequencing and assembly strategies, including long reads, linked-reads, and proximity data, have become available and fiscally feasible. Recently, new *B. napus* genomes using these technologies have been released to the public (Lee *et al.* 2020; Rousseau-Gueutin *et al.* 2020; Song *et al.* 2020). Concurrently, we generated a genomic assembly for a synthetic *B. napus,* similar to other recently released assemblies, that includes multiple previously unscaffolded sequences relative to the Darmor-bzh v4.1 assembly. In addition, this new assembly reveals shared and unique homoeologous exchange events compared with different lines of *B. napus*.

**Table 1.** Length of the 19 pseudomolecules, N50 of the pseudomolecules, total assembly N50, total pseudomolecule length, total number of scaffolds, and total assembly length.

The statistics of anchored chromosome length of 11 *B. napus* assemblies											
Chromosome	DaAe	Darmor-Bzh_V4.1	Darmor-Bzh_V10	Quinta	No2127	Westar	Tapidor	Gangan	Shengli	Zheyou7	ZS11
A01	30,963,416	23,267,856	32,958,928	34,049,429	34,418,524	34,072,108	33,385,296	36,624,584	36,358,838	34,646,657	38,004,428
A02	29,581,582	24,793,737	33,432,960	31,833,343	35,184,156	35,445,817	33,310,329	34,912,047	36,815,669	38,454,144	35,943,954
A03	38,724,999	29,767,490	39,685,748	44,229,005	44,024,636	45,938,915	45,374,774	42,633,929	40,627,569	46,180,332	44,868,710
A04	22,079,791	19,151,660	23,101,715	24,995,428	21,150,623	24,941,254	22,742,991	22,175,112	17,977,365	18,425,376	25,679,024
A05	29,228,566	23,067,598	42,112,164	40,081,539	42,988,262	43,898,138	41,389,975	37,886,159	42,946,112	44,454,297	45,991,561
A06	28,937,740	24,396,386	45,146,386	46,545,620	47,152,155	46,607,869	50,976,723	43,921,364	47,226,390	45,572,147	48,704,706
A07	28,277,616	24,006,521	29,390,523	29,069,541	29,386,911	32,489,665	31,880,213	37,845,779	32,247,771	28,254,355	32,302,721
A08	23,154,485	18,961,941	26,309,499	28,540,401	27,050,200	27,113,445	27,789,865	31,072,007	30,634,953	26,738,762	28,329,074
A09	45,044,935	33,865,340	53,549,826	69,118,063	68,282,462	67,849,243	63,820,574	69,520,783	69,970,591	64,447,344	65,862,748
A10	19,559,996	17,398,227	20,778,245	24,204,787	21,441,692	26,564,201	25,274,410	24,379,196	20,220,739	24,607,451	26,592,803
C01	51,431,623	38,829,317	48,239,358	49,844,256	48,500,495	55,568,513	50,976,155	56,937,391	53,237,480	50,906,239	57,880,920
C02	58,167,434	46,221,804	62,297,340	66,774,423	63,747,864	65,831,886	60,608,700	62,611,365	59,402,795	52,853,804	65,293,782
C03	74,222,928	60,573,394	73,669,886	79,398,332	71,696,257	72,844,319	77,070,395	79,770,199	73,374,125	75,927,486	79,061,710
C04	62,924,550	48,930,237	65,837,619	62,271,373	68,372,639	71,968,714	67,448,363	64,192,264	72,788,439	67,164,483	71,179,181
C05	56,537,224	43,185,227	56,382,805	57,344,646	59,684,040	59,594,324	55,324,214	59,804,570	55,526,125	60,477,792	59,550,008
C06	48,209,797	37,225,952	50,218,839	52,702,447	52,092,031	52,822,278	52,406,678	46,598,512	52,241,597	52,102,359	52,512,057
C07	54,958,258	44,770,477	55,656,957	59,821,549	59,911,058	55,243,557	61,376,810	60,504,736	57,612,675	60,142,069	60,986,212
C08	49,204,614	38,477,087	41,681,856	55,907,613	53,895,350	51,700,037	53,000,817	46,677,179	52,530,734	50,112,630	53,660,391
C09	64,956,572	48,508,220	66,465,249	63,235,258	61,068,053	64,409,414	66,565,279	63,602,739	56,715,283	65,173,479	68,416,614
**Pseudochromosome N50**	51,431,623	38,829,317	53,549,826	55,907,613	53,895,350	55,243,557	53,000,817	56,937,391	53,237,480	52,102,359	57,880,920
**Total assembly N50**	48,209,797	38,829,317	50,218,839	55,907,613	53,895,350	55,243,557	52,406,678	46,677,179	52,530,734	50,906,239	57,880,920
**Pseudochromosome**	816,166,126	645,398,471	866,915,903	919,967,053	910,047,408	934,903,697	920,722,561	921,669,915	908,455,250	906,641,206	960,820,604
**Total scaffolds**	3,164	41	237	3,722	3,733	3,458	3,566	4,930	3,802	4,990	3,332
**Total assembly**	1,001,499,700	923,795,763	923,795,763	1,004,262,373	1,012,393,425	1,008,283,116	1,014,411,283	1,034,272,646	1,002,553,391	1,016,238,606	1,010,887,456

## Materials and methods

### Creation of synthetic *B. napus*, Da-Ae

The synthetic *B. napus* cultivar Da-Ae (AACC, Korea patent number: 10-1432278-0000, 2014.08.13) used in this study was developed at FnPCo (South Korea) by crossing an inbred *B. rapa* (AA) Chinese cabbage (WC720) with an inbred *B. oleracea* (CC) red cabbage (BW716). After hybridization, the F_1_ underwent spontaneous chromosome doubling, producing a naturally occurring allotetraploid *B. napus (*AACC). The hybrid was self-fertilized, and 7 seeds were obtained and planted. Only 3 of the 7 plants germinated and flowered, with only one producing seeds. Progeny from this plant were then self-fertilized for 6 generations with the final generation being designated Da-Ae.

### Plant materials, DNA extraction, and library preparation

Three plant lines were sequenced in this study: the highly inbred Da-Ae, the male parent *B. rapa* (AA, WC720), and the female parent *B. oleracea* (CC, BW716). For each line, 100 seeds from a single plant were germinated and grown for 8–10 days. The resulting seedlings were pooled separately for each line and high molecular weight genomic DNA was extracted by Amplicon Express (Amplicon Express Inc., Pullman, WA, USA). The quality of the DNA collected from these 3 samples was assessed using a Bioanalyzer (Agilent Technologies, Inc., Santa Clara, CA, USA). A 10X Genomics library was prepared by the University of California, Davis (UCD) Genome Center. The resulting libraries were sequenced on an Illumina HiSeq X10 by Novogene (Novogene Corporation Inc., Sacramento, CA, USA) as 150 bp paired-end reads, producing ∼451, ∼380, and ∼380 million reads for Da-Ae, the male parent, and the female parent, respectively. An additional 10X Genomics library for Da-Ae was constructed by the UCD Genome Center using a library prep involving sonication, in contrast to the 10X Genomics library prep without sonication. This library was then sequenced on a HiSeq 4000 at the UCD Genome Center, producing ∼347 million 151 bp paired-end reads. For Pacific Biosciences (PacBio) sequencing, 32.9 µg high molecular weight DNA from Da-Ae was used for library construction and 19 SMRTcells were sequenced on a PacBio Sequel system (Pacific Biosciences, Menlo Park, CA, USA) at the UCD Genome Center, producing ∼6.6 million subreads with an average length of ∼11.2 kb. An additional 100 seeds from the same Da-Ae plant were grown to produce 4.5 g young leaf tissue, which was sent to Dovetail Genomics (Scotts Valley, CA, USA) for Hi-C library construction. The Hi-C library was then sequenced at the UCD Genome Center on an Illumina HiSeq 4000, producing ∼374 million 150-bp paired-end reads.

### Generation of 10X genomics assemblies

Initial assemblies of *B. napus* were generated using the default Supernova v1.1.5 pipeline (Weisenfeld *et al.* 2017) with an estimated genome size of 1.12 Gb. The 10X Genomics Da-Ae reads sequenced at the UCD Genome Center and Novogene (hereafter referred to as Da-Ae 10X Davis and Da-Ae 10X Novogene) were both independently assembled. The Da-Ae 10X Davis reads and the Da-Ae 10X Novogene reads resulted in near identical assemblies. As a result, only the Da-Ae 10X Davis reads were used in downstream Supernova assemblies. Upon the release of Supernova-2.0.0, the *B. rapa* 10X, *B. oleracea* 10X, and Da-Ae 10X Davis reads were each individually assembled using the newer software package. The number of reads required for 56X coverage was calculated using the formula genome size × 56/read length. The expected genome sizes used for *B. rapa*, *B. oleracea*, and *B. napus* were 530 Mb, 630 Mb, and 1.12 Gb, respectively. These values were then input to Supernova-2.0.0 using the –maxreads parameter. Scaffolds from these 3 new Supernova assemblies were later used to assess mis-assemblies in Dovetail scaffolding-based assemblies.

### Generation of PacBio assemblies

The PacBio reads were assembled using Canu version 1.6 (Koren *et al.* 2017) from Maryland Bioinformatics. Canu was configured for the 1.12 Gb genome size of *B. napus* and the reference parameter suggestions for high coverage, polyploid organisms, which are corrected ErrorRate = 0.040 and corOutCoverage = 200. The Canu pipeline consisted of 3 separate steps: correction, trimming, and assembly.

### Polishing of PacBio assemblies

Polishing was performed to improve the quality of the Canu Da-Ae assembly. Polishing was completed using the 10X Da-Ae Davis reads and the Broad Institute’s program Pilon v.1.22 (Walker *et al.* 2014). Following the guidelines from 10X Genomics, 23 bp at the start of read 1 and the first base pair of read 2 were removed using Trimmomatic v.0.33 (Bolger *et al.* 2014) in order to remove the 10X barcodes and the initial base of read 2 that is often low quality. The trimmed reads were then mapped to the Canu Da-Ae assembly using bwa version 0.7.16a (Li and Durbin 2009). The assembly and the mapped read files were fed into Pilon. After polishing, the assembly had approximately the same size and N_50_ as its unpolished counterpart.

### Hi-C scaffolding of PacBio assemblies

The Canu Da-Ae assembly and the Hi-C reads sequenced at the UCD Genome Center were sent to Dovetail Genomics for scaffolding. The assembly and the Hi-C reads were run through Dovetail's proprietary HiRise pipeline, where the individual contigs were scaffolded to create chromosome-scale scaffolds.

### Analysis of Hi-C results

The N_50_, assembly size, and BUSCO scores of the HiRise scaffolded assembly were measured. All scaffolds from the HiRise-generated assembly were compared with the chromosomes of the publicly available Darmor-bzh v4.1 genome hosted by the Brassica database (BRAD) (Cheng *et al.* 2011). The scaffolds from the HiRise-generated assembly were independently aligned to the chromosomes of Darmor-bzh v4.1 using Nucmer with the parameters –maxmatch -l 100 -c 500. The alignments were filtered for quality and all scaffolds 1 Mb or greater were plotted (see Supplementary Fig. 1 in Supplementary File 1). If a scaffold aligned best to 1 reference chromosome, it was assigned a name based on its alignment. All remaining scaffolds in the assembly were not renamed and retained their HiRise-designated sequence IDs. A Hi-C contact map was also generated to access the quality of the assembly. The Da-Ae Hi-C reads were mapped to the Canu Da-Ae assembly using the Arima mapping pipeline developed by Arima Genomics (https://github.com/ArimaGenomics/mapping_pipeline). The BAM file was then converted to a 4dn style pairs file using the *bam2pairs* utility script which is part of the Pairix program suite (https://github.com/4dn-dcic/pairix). The resulting pairs file was then input into Juicer pre-program to generate a .hic file (Durand *et al.* 2016). Visualization of the .hic file was then completed using Juicebox (Robinson *et al.* 2018).

### Assessing discrepancies between the Canu Da-Ae assembly and the public reference assembly

The 21 largest scaffolds in the assembly were independently compared with their corresponding Darmor-bzh v4.1 chromosomes (Darmor-bzh v10 was not available at this time). Regions of discrepancy between the assembly and the reference assembly were identified. The validity of each discrepancy was then tested by aligning PacBio reads and 10X ancestral parent scaffolds to the Canu Da-Ae assembly. The PacBio reads were aligned using BLASR (Chaisson and Tesler 2012) with a minimum subread length of 10 kb. The 10X ancestral parent scaffolds were aligned using nucmer from the MUMmer software suite (Marçais *et al.* 2018). If the region of discrepancy in the assembly had substantial support from the mapped reads and scaffolds, substantial meaning the mapped reads and/or scaffolds spanned the region of the discrepancy and aligned with the Da-Ae assembly, the discrepancy was considered a true difference between our assembly and the Darmor-bzh v4.1 assembly; thus, it was retained. If there was no support, or the mapped reads and scaffolds disagreed with the Canu Da-Ae assembly, the region of discrepancy was considered a likely error and altered to match Darmor-bzh v4.1. All alterations performed were simple sequence flips to fix assembly inversions. All inversions, except one, were almost exactly encapsulated by the contig boundaries of a scaffold (see Supplementary Table 1 in Supplementary File 1). After all identified discrepancies had been addressed, the assembly was considered final. After Darmor-bzh v10 was released, we compared DArmor-bzh v4.1 and v10 and found them to be essentially colinear. The one exception was an inversion on C07, a region where Da-Ae also showed a supported inversion relative to Darmor-bzh v4.1 (that we had not changed). A Hi-C contact map (see Supplementary Fig. 2 in Supplementary File 1) generated by juicer (Durand *et al.* 2016) and juicebox (Robinson *et al.* 2018) supports our assembly.

### Transcriptome assembly and structural annotation of novel transcripts

RNA-seq reads from 13 RNA-sequencing libraries generated from 5 tissues (young leaf, flower, bolting tissue, 1 cm silique, and 5 cm silique) of Da-Ae (Li *et al.* 2018) were used for transcriptome assembly and annotation. The raw sequencing data were preprocessed and mapped to the published genome sequence of Darmor-bzh (*B. napus* genome v4.1) as described in Li *et al*. (2018). The mapped reads were then assembled to transcripts using Cufflinks v2.2.1 (Trapnell *et al.* 2010) with the help of reference annotations. The output GTF files generated by Cufflinks were fed to Cuffmerge and then compared with the annotations from the reference assembly using Cuffcompare. From the output file, transcripts with code “u” were considered novel. Redundant isoforms among these novel transcripts were removed using CAP3 (Huang and Madan 1999), and only transcripts with open-reading frames detected using TransDecoder (Haas *et al.* 2013) were retained for the next step. For de novo assembly, post-processed high-quality reads were pooled together and assembled using Trinity (Grabherr *et al.* 2011) set to default parameters. The abundance of transcripts was estimated using the Kallisto (Bray *et al.* 2016) method implemented in the Trinity pipeline, and those with <1 transcript per kilobase million were removed. Transcripts with detected open-reading frames were aligned to the Darmor-bzh coding sequences (CDS) using BLASTN (Altschul *et al*. 1990) with an *E*-value cutoff of 1e-6, and those with high identity (≥95%) to Darmor-bzh CDS were filtered. An additional BLASTX search was conducted against NCBI non-redundant protein database using *E*-value 1e-6 to remove transcripts with no homology to known plant genes. The resulting assembly from reference-based and de novo methods were combined for structural annotation using DAMMIT (Scott 2016) with default parameters to generate the final GFF3 file. BUSCO scores for the final assembly were calculated to assess transcriptome completeness (Cantarel *et al.* 2008).

### Annotation using MAKER

Annotation was performed using MAKER v.3.01.02-beta (Cantarel *et al.* 2008, Campbell, Holt, *et al.* 2014). Prior to running the MAKER pipeline, a custom repeat library was constructed using the MAKER-P Repeat Library Construction-Advanced (Campbell, Law, *et al.* 2014; see Supplementary Table 2 in Supplementary File 1). MAKER was run with the following parameters: the CDS transcripts from the Darmor-bzh v4.1 assembly (Chalhoub *et al.* 2014), Darmor-bzh v10 assembly (Rousseau-Gueutin *et al.* 2020), and the 8 *B. napus* assemblies (ZS11, Westar, No2127, Zheyou7, Gangan, Shengli, Tapidor, and Quinta) from Song *et al.* (2020); the previously identified novel transcripts were used as expressed sequence tag evidence. The peptide sequences from each *B. napus* assembly mentioned above as well as *B. oleracea* HDEM*, B. rapa* Z1 v2 downloaded from genoscope.cns.fr, and the *Arabidopsis thaliana* Araport11 peptides downloaded from the TAIR Project (Berardini *et al.* 2015) were used as evidence for protein homology. MAKER parameters that were modified included the following: A custom Augustus gene prediction species model of Da-Ae created using BUSCO v3.0.2 with the long parameter was used as the model species for Augustus; repeat library was set to the custom repeat library we constructed using the MAKER-P Repeat Library Construction-Advanced protocol; est2genome was set to 1; protein2genome was set to 1. All other parameters not listed above were left as the MAKER defaults. Due to an unresolved bioinformatics issue, 10 kb of chrC01 sequence starting at 47,446,387 had to be masked with N before MAKER would run to completion.

Once annotation of each chromosome was completed, the MAKER proteins were compared with the Uniref90 protein set using BLASTP. Protein domains were then identified using InterProScan on the MAKER predicted proteins. Using accessory scripts provided with MAKER, the MAKER genes were then renamed with the prefix “Bna,” the suffix “Da-Ae,” and the BLASTP and InterProScan results were integrated into the GFF annotation files. Finally, the annotations were filtered to remove any annotation that contained an Annotation Edit Distance score >0.5. The cutoff of 0.5 was selected based on the recommendation listed in Campbell, Holt, *et al*. (2014).

### Analysis of homoeologous exchange between subgenomes

We examined homoeologous exchange using 2 methods: synteny analysis and read coverage. For synteny analysis, we first identified “trusted” syntenic regions of the A and C subgenomes by performing a nucmer (Marçais *et al.* 2018) alignment of *B. rapa* (A chromosomes) and *B. oleracea* (C chromosomes) assemblies; hits were filtered to require >85% identity for >1,000 bp. We next used nucmer to align each *B. napus* assembly in our comparison (Da-Ae, Darmor-BZH_V10, GanganF73, No2127, QuintaA, Shengli3, Tapidor, Westar, Zheyou73, and ZS11) to an in silico *B. napus* genome constructed by combining both the *B. rapa* (Istace *et al.* 2021) and the *B. oleracea* (Belser *et al.* 2018) chromosomes (hereafter referred to as “ancestral”). Candidate homoeologous exchange regions were defined as those where the sequence was from an “A” subgenome in the *B. napus* assembly but had its highest hit to a “C” region in the “ancestral” assembly or vice versa. Candidate homoeologous exchange regions were filtered to retain only those with >90% identity, a length of >100 bp, and overlap with “trusted” syntenic regions in the ancestral assembly (to eliminate false positives that could be caused by assembly gaps in *B. rapa* or *B. oleracea*). All nucmer runs used version 4.0.0 with default parameters; all nucmer results were filtered to retain the one best alignment using the delta-filter program with option “-1”.

When sequence reads from one genome are mapped to an assembly from a different genome, differences in homoeologous exchange between the two genomes will lead to decreased or increased read coverage. To utilize this kind of information, we performed a coverage analysis using the 10X Da-Ae Davis reads. All reads were trimmed for quality using Trimmomatic and the adapter sequences were removed with the parameters ILLUMINACLIP:adapters.fa:2:30:10 LEADING:3 TRAILING:3 SLIDINGWINDOW:4:15 MINLEN:36 before being mapped with BWA to the in silico “ancestral” *B. napus* genome described above. To find possible sites of homoeologous exchange, we first filtered the 10X Da-Ae Davis reads to retain those that could reliably be described as coming from either the A or C subgenome (i.e. those with unique and trustworthy mapping locations). To do so, the alignment file was filtered to only contain alignments that had a MAPQ of 5 or greater, were properly paired, had no supplementary alignments, and were primary alignments. Reads that passed these filters were then mapped to 9 *B. napus* genomes. The coverageBed function from bedtools2 v2.29.2 (Quinlan and Hall 2010) was then used to calculate the coverage across the genomes and the coverage of the individual potential genes previously identified. The alternate mapping sites were also captured using the “XA” tag from the bwa output. Using edit distance as a filtering parameter, alternate mapping sites that had an edit distance equal to or less than the primary alignment's edit distance were added to the coverage calculation. To calculate coverage across the genomes, median coverage in a window size of 100 kb with a step size of 20 kb was used. The calculated coverages were standardized based on the genome-wide average using R (R Core Team 2021). Prior to standardization, regions that contained ≥10X mean coverage of their chromosome were removed from further analysis. The coverages were then plotted to identify regions across the genome with higher or lower than average coverage. Coverages were plotted using ggplot2 (Wickham 2009) in R. Plots combining the coverage and synteny analyses were plotted using a modified version of the plotsr program (Goel and Schneeberger 2022).

Annotation of genes in shared homoeologous exchange regions was done by using BLASTP to query an *Arabidopsis* ARAPORT11 protein database with *B. rapa* or *B. oleracea* protein sequences at phytozome (Goodstein *et al.* 2012), keeping the one best hit, and downloading ARAPORT11 annotations from phytozome.

## Results

**Fig. 1.**
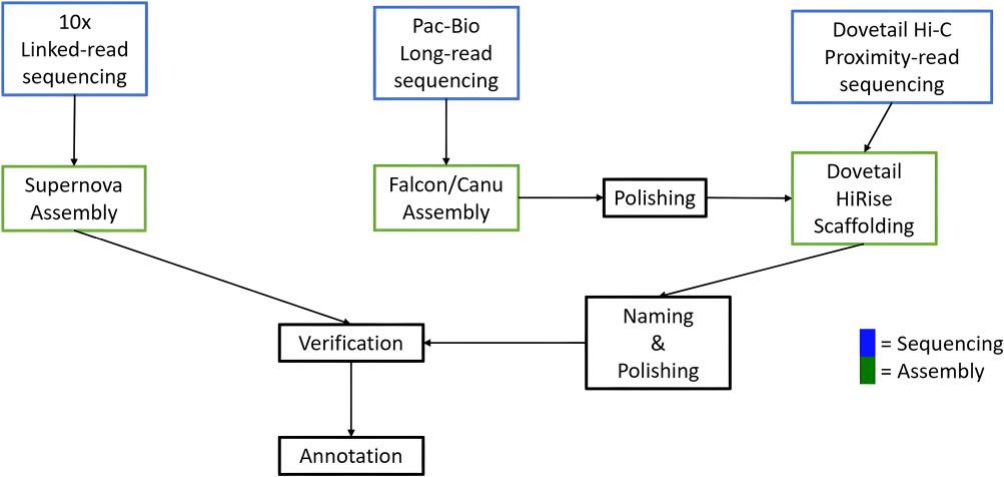
Genome assembly and annotation strategy.

To develop a high-quality assembly of this new, synthetic *B. napus,* we took advantage of contemporary technologies by using a combination of 10X Genomics, Pacific Biosciences, and Dovetail/Hi-C methods (Fig. 1). The application of each is described in turn below, followed by the results of the annotation and homoeologous exchange analysis.

### Supernova assemblies

The Da-Ae 10X Davis reads were assembled with Supernova v2.0.0. The assembly had a length of 918 Mb and an N_50_ of 1.5 Mb. Notably, the BUSCO scores of this new assembly approached the scores of the Darmor-bzh v4.1. The 10X reads for both *B. rapa* and *B. oleracea* assembled using Supernova v2.0.0 also showed promising results. Both assemblies had N_50_ values over 2 Mb and consisted of <20,000 scaffolds. Although all assemblies were smaller than the expected genome sizes, they were all on par with the sizes of the public references. The assembly metrics and BUSCO scores supported the use of the assembly scaffolds in the manual curation of the subsequent *B. napus* Da-Ae assembly.

### PacBio assembly and dovetail scaffolding

Da-Ae PacBio reads were assembled with Canu and polished with Pilon. This Pilon-polished Canu Da-Ae assembly was then scaffolded using the HiRise pipeline by Dovetail Genomics. After HiRise scaffolding, the Canu Da-Ae assembly showed a large increase in N_50_ from 1.59 to 42.79 Mb, and had 3,190 scaffolds. Twenty-three of the scaffolds were >1 Mb, with the largest being 74.2 Mb (see Supplementary Table 3 in Supplementary File 1). Regarding BUSCO scores, the scaffolding caused the single to duplicate ratio to increase in the Canu Da-Ae assembly while the percentage of complete BUSCO scores, 98.6%, did not change in the Canu Da-Ae assembly (see Supplementary Table 3 in Supplementary File 1).

### Assigning scaffolds to chromosomes

To assign the scaffolds to the established chromosomes, the assembly was aligned to the Darmor-bzh v4.1 assembly using Nucmer. The 19 Darmor-bzh v4.1 chromosomes were covered by the 21 largest Canu scaffolds; 17 spanned the full length of their sister Darmor-bzh scaffold, while the remaining 4 scaffolds had to be concatenated into pairs to span ChrC06 and ChrC07 (see Supplementary Fig. 1 in Supplementary File 1). Names were then assigned to the scaffolds based on which Darmor-bzh chromosome they aligned to.

### Assembly discrepancies

**Fig. 2.**
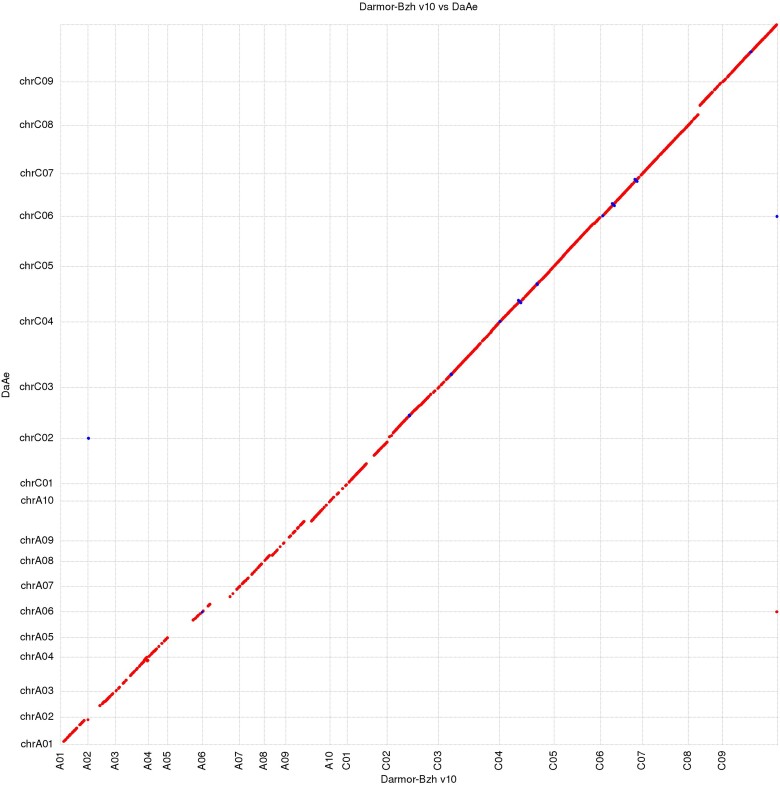
Nucmer plot of the final Da-Ae assembly aligned to the Darmor-bzh v10 reference. A total of 19 final assembly pseudomolecules are aligned to 19 reference pseudomolecules. Red indicates an alignment in the forward direction and blue indicates an alignment in the reverse direction.

Comparison of the Canu Da-Ae assembly to the Darmor-bzh v4.1 assembly revealed 24 assembly discrepancies (see Supplementary Table 1 in Supplementary File 1). These discrepancies included inversions, lack of contiguity, and introduction of new sequence. To assess the validity of these discrepancies, both the parental 10X scaffolds and the PacBio reads were mapped to the Canu Da-Ae assembly. In 15 of the 24 discrepancies, the Canu Da-Ae assembly was supported by either read mapping or scaffold evidence. In ChrC06 and ChrC07, two scaffolds spanned the whole-reference chromosome but failed to be scaffolded together. These scaffolds were joined with 100 Ns to signify a scaffolding gap and were then able to span the entire Darmor-bzh v4.1 chromosome as one scaffold. In 6 cases, the Canu Da-Ae assembly had unsupported inversions with 4 of the inversions spanning from one scaffold gap to another scaffold gap. For each case, the sequence was inverted to match the Darmor-bzh v4.1 assembly. The most prominent discrepancy occurred on ChrA05. Alignment to Darmor-bzh v4.1 suggested that both chromosome arms were inverted at their junction with the centromere. As there was no read or scaffolding evidence to support this, both chromosome arms were inverted to match Darmor-bzh. Although our ChrA05 now agrees with the Darmor-bzh v4.1 assembly, the orientation and centromeric region remains questionable. After all discrepancies were addressed, the assembly was deemed final and annotation began. Darmor-bzh v10 was released after our assembly was finalized. Darmor-bzh v4.1 and v10 are nearly colinear. In the one place where they are not, C07, Da-Ae matches v10, so there was no need to update our assembly. Figure 2 provides a synteny plot of the final Da-Ae assembly against Darmor-bzh v10.

### Annotation

MAKER analysis of the Da-Ae assembly predicted 125,439 protein-coding genes after filtering, compared with the 101,400 and 108,190 genes annotated in the Darmor-bzh v4.1 and v10 assemblies. To explore these differences, we determined the location of the predicted genes in their respective assemblies. Da-Ae contains more gene models than Darmor-bzh v4.1 and v10, with 123,488 of the Da-Ae gene models being present on its 19 pseudomolecules compared with Darmor-bzh v4.1 and v10 which contain 80,927 and 106,885 gene models on their 19 pseudomolecules, respectively. These discrepancies could be due to the differences in length of time since polyploidization. Since Da-Ae is a new synthetic, it has had much less time for gene loss after the polyploidization event.

### Final assembly comparison

The final Da-Ae assembly improves upon the Darmor-bzh v4.1 assembly by a number of criteria (Tables 1 and 2). Comparing the full assemblies and the pseudomolecule assemblies, respectively, the N50 is 24–32% longer, and there are 36–47% more unambiguous bases incorporated into the Da-Ae assembly (Table 1). When compared with the Darmor-bzh v10 assembly, the full Da-Ae assembly and the pseudomolecule assembly each have 4% shorter N50s. However, the full Da-Ae assembly has 12% more unambiguous bases than Darmor-bzh v10, while the pseudomolecule Da-Ae assembly has 4% fewer unambiguous bases than Darmor-bzh v10 (Table 1). When comparing BUSCO scores using the brassicales_odb10 data set, both the Da-Ae assembly and the Darmor-bzh v10 assemblies had BUSCO complete scores of 98.5%, while Darmor-bzh v4.1 had a slightly lower score of 98.2%. Both Darmor-bzh assemblies had a higher percentage of complete single-copy BUSCO score, whereas the Da-Ae assembly had a higher percentage of duplicated BUSCO scores.

**Table 2.** Percentages of BUSCO scores.

The BUSCO (%) statistics of 11 *B. napus* assemblies						
Assembly	Complete BUSCO scores	Complete single-copy BUSCO scores	Complete duplicated BUSCO scores	Fragmented BUSCO scores	Missing BUSCO scores	# BUSCO scores
DaAe	98.5	18.0	80.5	0.2	1.3	4,596
Darmor-Bzh_V4.1	98.2	20.6	77.6	0.2	1.6	4,596
Darmor-Bzh_V10	98.5	19.6	78.9	0.1	1.4	4,596
Quinta	98.7	20.1	78.6	0.0	1.3	4,596
No2127	98.4	25.1	73.3	0.2	1.4	4,596
Westar	98.7	19.9	78.8	0.0	1.3	4,596
Tapidor	98.5	21.7	76.8	0.1	1.4	4,596
Gangan	98.5	21.7	76.8	0.1	1.4	4,596
Shengli	98.4	22.1	76.3	0.1	1.5	4,596
Zheyou	98.5	21.6	76.9	0.0	1.5	4,596
ZS11	98.5	19.9	78.6	0.1	1.4	4,596

BUSCO score percentages were calculated using the brassicales_odb10 data set, which contains 4,596 BUSCO scores.

### Genome completeness analysis

**Fig. 3.**
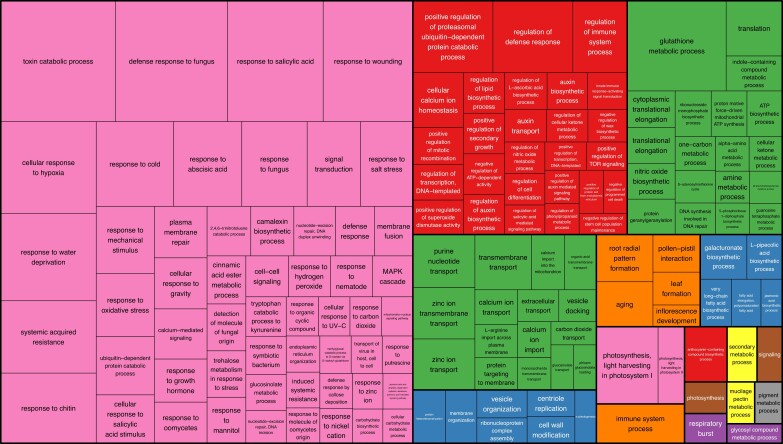
Tree map displaying over-represented BP:GO terms in the set of unigene sequences not found in Da-Ae or Darmor-bzhv10.

**Fig. 4.**
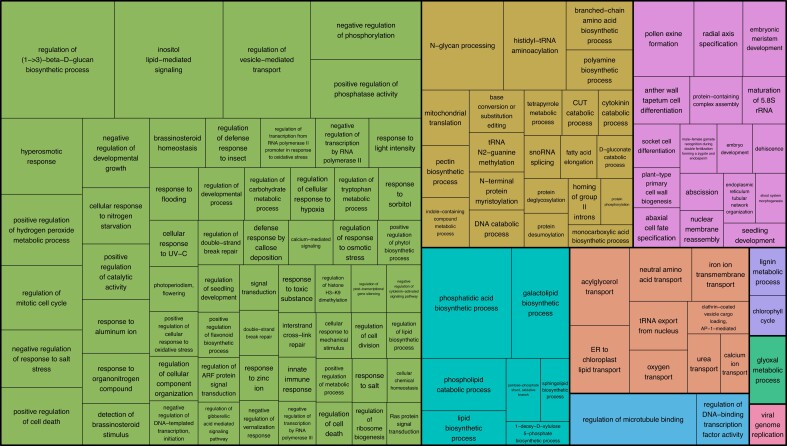
Tree map displaying over-represented BP:GO terms in the set of unigene sequences found in Da-Ae but not Darmor-bzh v10 and vice versa.

Genome completeness of Da-Ae and Darmor-bzh v10 was analyzed using the public unigene set of 133,127 *Brassica* sequences. Of the 133,127 sequences, 116,897 (87.81%) were present in the pseudomolecules of both genomes. Overall Darmor-bzh v10 contained the most unigene sequences, 118,199, with Da-Ae a close second with 118,193 unigene sequences. A total of 13,632 (10.24%) were missing from both genomes. To determine if there were classes of genes that were deleted/missing in these genomes, we looked for enriched GO terms among the set of genes missing from the two genomes. Among the enriched categories, enrichment for genes involved in responses to biotic and abiotic stressors was particularly noticeable, as seen in the pink box in the left of Fig. 3 (Supek *et al.* 2011). We also looked for unigenes present in Da-Ae but not in Darmor-bzh v10 and vice versa. Here, among the enriched categories, we noted an enrichment for genes involved in very long-chain fatty acid metabolism, perhaps reflecting different breeding selection targets for these oilseed crops, as seen in the teal box in the bottom middle of the treemap (Fig. 4).

### Homoeologous exchange

Homoeologous exchange is the exchange of genetic material from one subgenome to the other. This could result in the conversion of an A subgenome gene to a C subgenome gene or vice versa. Because *B. napus* is an allotetraploid containing two diploid subgenomes, A and C, homoeologous exchange can result in homoeolog ratios of 2:2, 3:1, or 4:0, corresponding to reciprocal, partial, or complete conversions, respectively. We used two criteria to identify and characterize candidate homoeologous exchange regions: (1) a synteny analysis in which the *B. napus* genome assemblies were aligned to concatenated *B. rapa* and *B. oleracea* genomes, as proxies for the ancestral A and C subgenomes; (2) a coverage analysis in which we looked for regions of decreased or increased coverage that would result when two *B. napus* genomes had different homoeologous exchange (see Materials and methods).

For the coverage analysis, we examined read coverage of Da-Ae when mapped to itself, a pseudo “ancestral” genome of concatenated *B. rapa* and *B. oleracea*, and 9 existing *B. napus* assemblies (see Supplementary Figs. 3–21 in Supplementary File 1). Note that in these plots, average coverage is normalized to “1”; a partial conversion (one but not both homologs) will result in readings of ∼0.5 and ∼1.5, whereas a complete conversion will give coverages of ∼0 and ∼2 on this scale. Each chromosome shows a region of coverage elevated to 4X or higher, likely representing centromeric regions where the repeats are collapsed in the assembly. In addition, we see numerous regions with coverage in the 0, 0.5, 1.5, or 2X range.

**Fig. 5.**
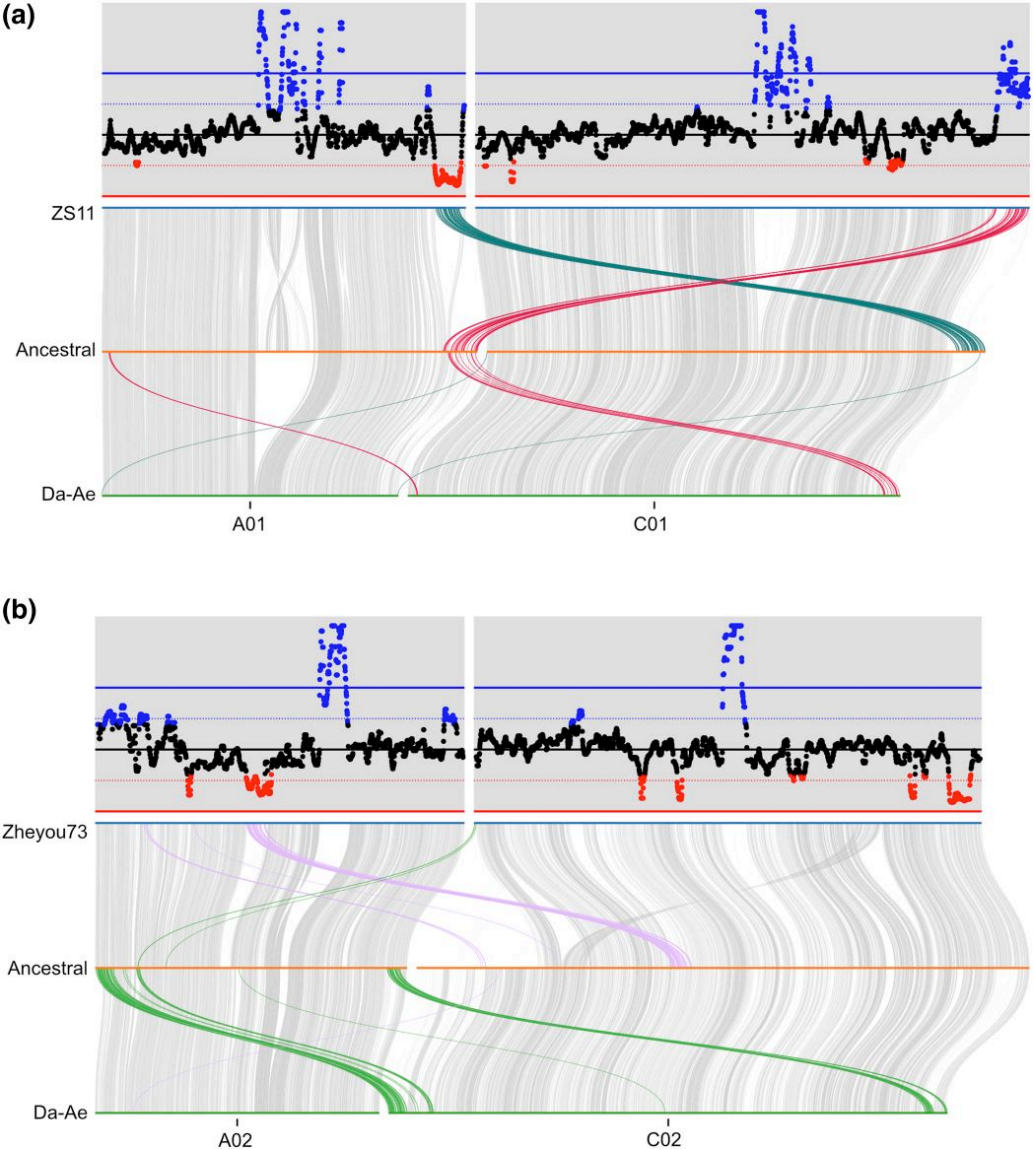
Examples of homoeologous exchange and coverage of Da-Ae reads when mapped to other genomes. The upper parts of each panel show the coverage of Da-Ae reads when mapped to ZS11 (a) or Zheyo73 (b), while the lower parts show syntenic and homoeologous exchange regions. a) ZS11 has a reciprocal exchange between the right ends of A01 and C01, whereas DaAe has a replacement of C01 with A01 in this region. b) Zheyou73 and DaAe show several non-overlapping, non-reciprocal exchanges between A02 and C02.

To determine if any of regions with increased or decreased coverage might result from homoeologous exchange vs the aneuploidy that is common in nascent synthetic lines (Xiong *et al.* 2011; Ferreira de Carvalho *et al.* 2021), we plotted the coverage and synteny analysis together (see Supplementary Figs. 22–32 in Supplementary File 1). In many cases, the change in coverage is due to homoeologous exchange. For example, examining ZS11 and Da-Ae reveals that ZS11 had a reciprocal exchange between the right-hand sides of A01 and C01, whereas in Da-Ae this region of C01 was converted to A01 (Fig. 5a). As a consequence, there is low Da-Ae coverage at the end of ZS11 A01 and high coverage at the end of ZS11 C01 (since that region corresponds to ancestral A01 and Da-Ae has two homoeologs matching A01 in this region). Comparing Zheyou73 and Da-Ae A02 and C02 reveals that both ends of Da-Ae C02 have been converted to A02 and a region in the middle of Zheyou73 A02 has been converted to C02, with read coverage changing as expected (Fig. 5b). Other regions with increased or decreased coverage but no evidence of homoeologous exchange could result from insertion/deletion differences between the genomes, aneuploidy, or incomplete genome assemblies.

**Fig. 6.**
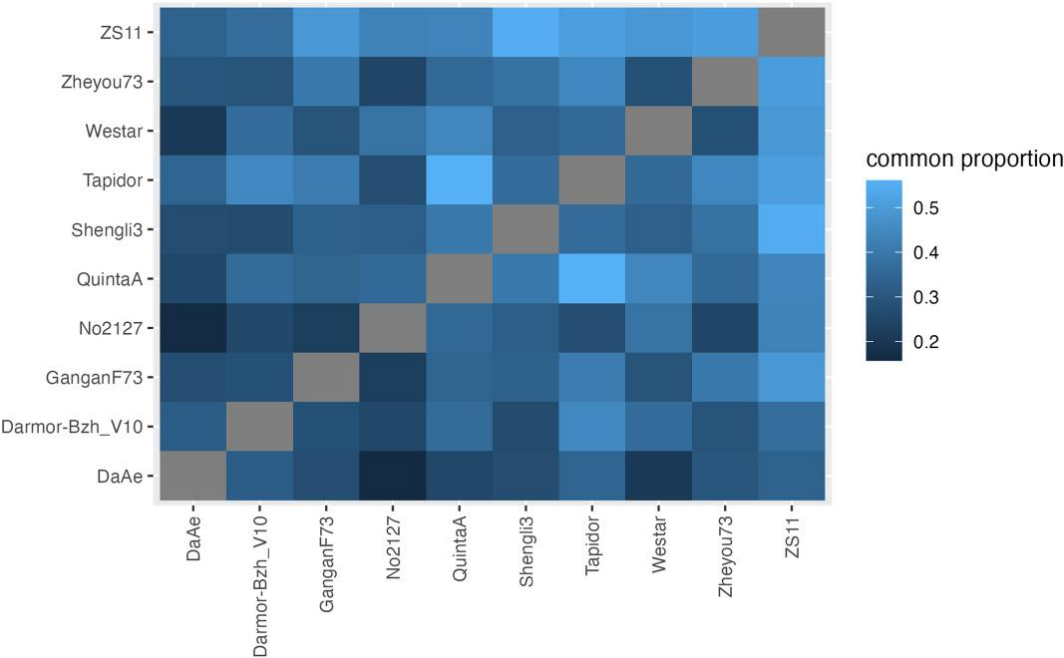
Proportion of common homoeologous exchange regions between genomes. Each tile shows the amount of shared homoeologous exchange regions between the genomes, proportional to the pairwise minimum homoeologous shared amount.

The synteny plots (see Supplementary Figs. 22–32 in Supplementary File 1) reveal that there are numerous regions where homoeologous exchange has occurred in the same place in different genomes. Since Da-Ae and No2127 are independent synthetic *B. napus* lines, this suggests that there are hotspots of homoeologous exchange. Figure 6 shows the similarity in homoeologous exchange regions across the varieties; as expected, the two synthetic varieties, Da-Ae and No2127 are the most dissimilar from the other varieties. We next asked if there were any homoeologous exchange regions shared among all varieties. We found 31 homoeologous exchange regions encompassing a total of 39 kb that were common across all varieties. This is more overlap than predicted by chance; based on the proportion of each genome involved in homoeologous exchange we would expect zero bases to be common across all varieties. There are a total of 16 genes in the conserved exchange regions, 14 of which had strong homologs in the *Arabidopsis* genome (see Supplementary Table 4 in Supplementary File 1). Of these 14, one, *BolC8t52214H*, is a nucleotide binding site leucine-rich repeat protein whose closest *Arabidopsis* homolog is *AT1G12210* or *RPS5-LIKE 1*, a close paralog of the defense R gene *RPS5*. Two other genes have leucine-rich repeats, although their relationship to plant immunity is less clear.

## Discussion

Since the release of the first reference genome (Chalhoub *et al.* 2014), multiple research groups have released genome assemblies of different *B. napus* cultivars, analyzed homoeologous exchange, and identified quantitative trait loci related to key agricultural traits (Wang *et al.* 2015; Bayer *et al.* 2017; Samans *et al.* 2017; Stein *et al.* 2017; Song *et al.* 2020; Rousseau-Gueutin *et al.* 2020; Boideau *et al.* 2022). These efforts all contribute to untangling the genome biology of *B. napus* that will one day be combined to create a species-wide pan-genome.

The first *B. napus* reference was assembled and released during a time when sequencing technologies from PacBio, 10X Genomics, and Dovetail Genomics were in their infancy and/or not fiscally feasible for most research groups. As a result, the first release of the *B. napus* genome was not able to benefit from the analytical power of these technologies. This is reflected in the assembly size of the Darmor-bzh V4.1 genome (Chalhoub *et al.* 2014). Although the expected size of the *B. napus* genome is over 1 Gb, the Darmor-bzh V4.1 genome assembly is only ∼850 Mb, of which 650 Mb is contained in 19 chromosome-scale pseudomolecule scaffolds. By using a recently created synthetic *B. napus*, *Da-Ae*, along with long-read, linked-read, and proximity ligation technologies, we were able to generate a new synthetic *B. napus* genome that exceeded the first high-quality reference genome by several metrics and is on par with more contemporary assemblies. Our assembly of Da-Ae is over 1 Gb, with >800 Mb contained within 19 chromosome-scale pseudomolecule scaffolds. While our assembly is larger compared with both the Darmor-bzh V4.1 and v10 assemblies, it still maintains a high level of sequence collinearity with the two Darmor-bzh assemblies. On a gene level, the Darmor-bzh v4.1 and v10 references have fewer annotated genes than our assembly. The differences in the high-quality assemblies may reflect differences in genome content due to the synthetic Da-Ae having had fewer generations in which to “purge” extra material (resulting in, for example, larger number of bases and more duplicated BUSCO scores) or could reflect differences resulting from the assembly process. It is not possible to distinguish between these causes with our available data. The improved assembly enabled by third generation sequencing technologies will serve as an excellent resource for *B. napus* geneticists and scientists aiming to identify genes underlying agronomic traits.

Homoeologous exchange is a biological process observed in allopolyploids, like *B. napus*, where highly similar yet different regions of the two diploid subgenomes exchange genetic material with one another. The result is new chromosome structures that, while being primarily composed of one ancestral genome, now also contain regions belonging to a different ancestral genome. To investigate the occurrence of homoeologous exchange in Da-Ae, we investigated both genome coverage and synteny across the genomes of *B. napus* Da-Ae, and 9 other cultivars. Our results indicate that homoeologous exchange has occurred in both small and large regions throughout the whole genome. Each cultivar of *B. napus* had many unique homoeologous exchange events. More surprising was that there are multiple regions of homoeologous exchange that are shared among the *B. napus* cultivars. It is possible that these homoeologous exchange regions are shared among multiple varieties because specific combinations of homoeologous genes affect plant fitness or agroeconomic traits. Alternatively, these sites could be shared because sequence homology and chromosome topology favors recombination at these sites. These findings further build upon the previous work done to identify hotspot regions (Higgins *et al.* 2018).

In conclusion, using several sequencing technologies, we created a genome assembly similar in quality to other recently published assemblies that used third generation sequencing, allowing for an improvement upon the original Darmor-bzh v4.1 published assembly. We also identified potential hotspots of homoeologous exchange along with single-copy BUSCO score that are shared among different cultivars of *B. napus*. Our assembly and analysis of Da-Ae is another step forward toward the realization of a pan-genome for *B. napus*.

## Supplementary Material

jkad026_Supplementary_Data

## Data Availability

All raw reads and the nuclear genome assembly and annotation are available at NCBI under BioProject PRJNA627442. Analysis code is available at https://github.com/MaloofLab/Davis_B_napus_assembly_2023. Supplemental material is available at figshare: https://doi.org/10.25387/g3.21909729.
