# Supplementary material for: Whole-genome sequence of synthetically derived Brassica napus inbred cultivar Da-Ae
Source: G3 (Bethesda). 2023 Feb 1;13(4):jkad026. doi: 10.1093/g3journal/jkad026 (PMC10085753; doi:10.1093/g3journal/jkad026)
Supplement: jkad026_Supplementary_Data [file jkad026_supplementary_data.docx]

**Supplemental Materials**


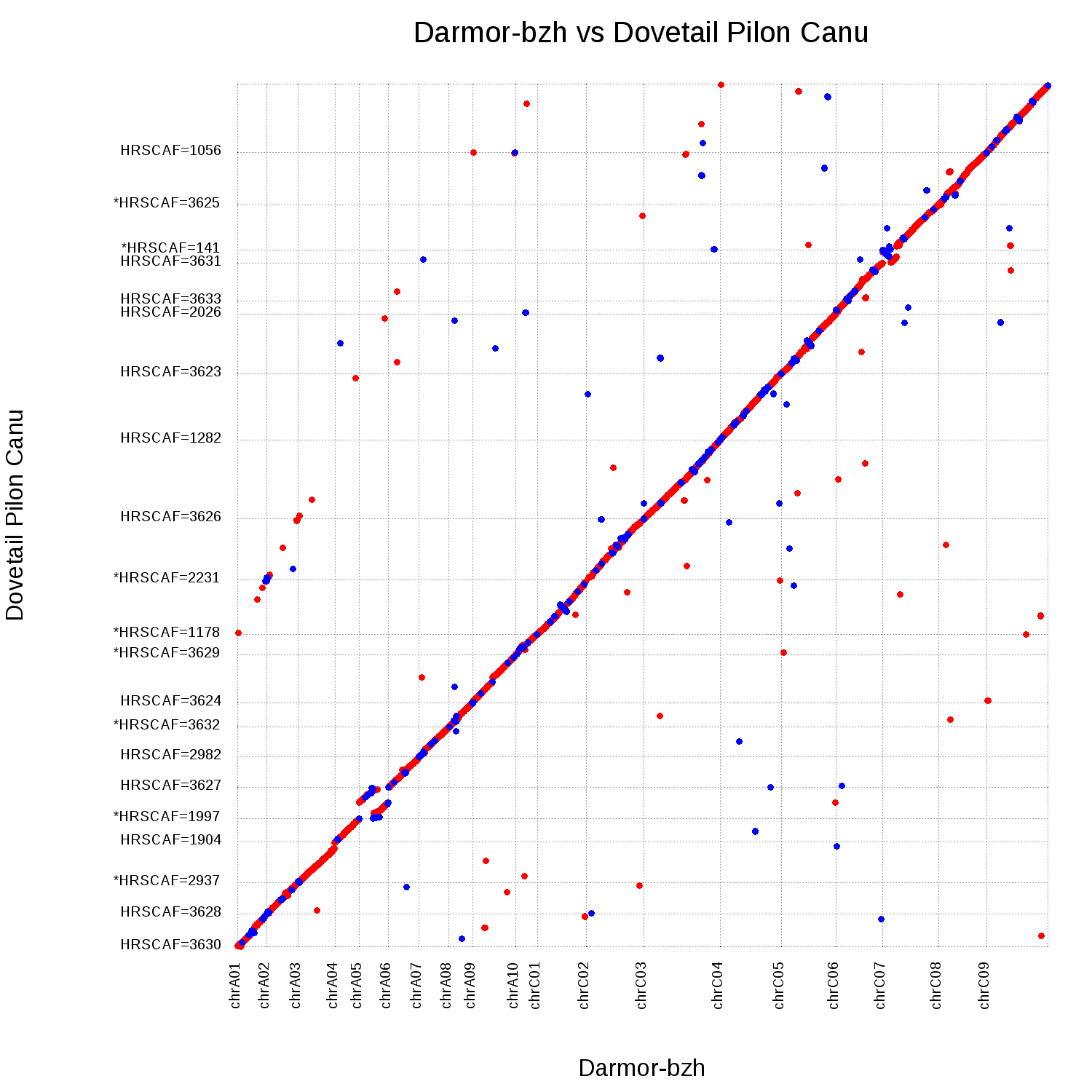


**Figure S1**. Nucmer plot of Dovetail_Pilon_Canu aligned to Darmor-bzh v 4.1 chromosomes. All sequences aligned are 1 Mbp or greater. A total of 21 Dovetail_Pilon_Canu scaffolds are aligned to 19 reference chromosomes. Red indicates an alignment in the forward direction and blue indicates an alignment in the reverse direction.


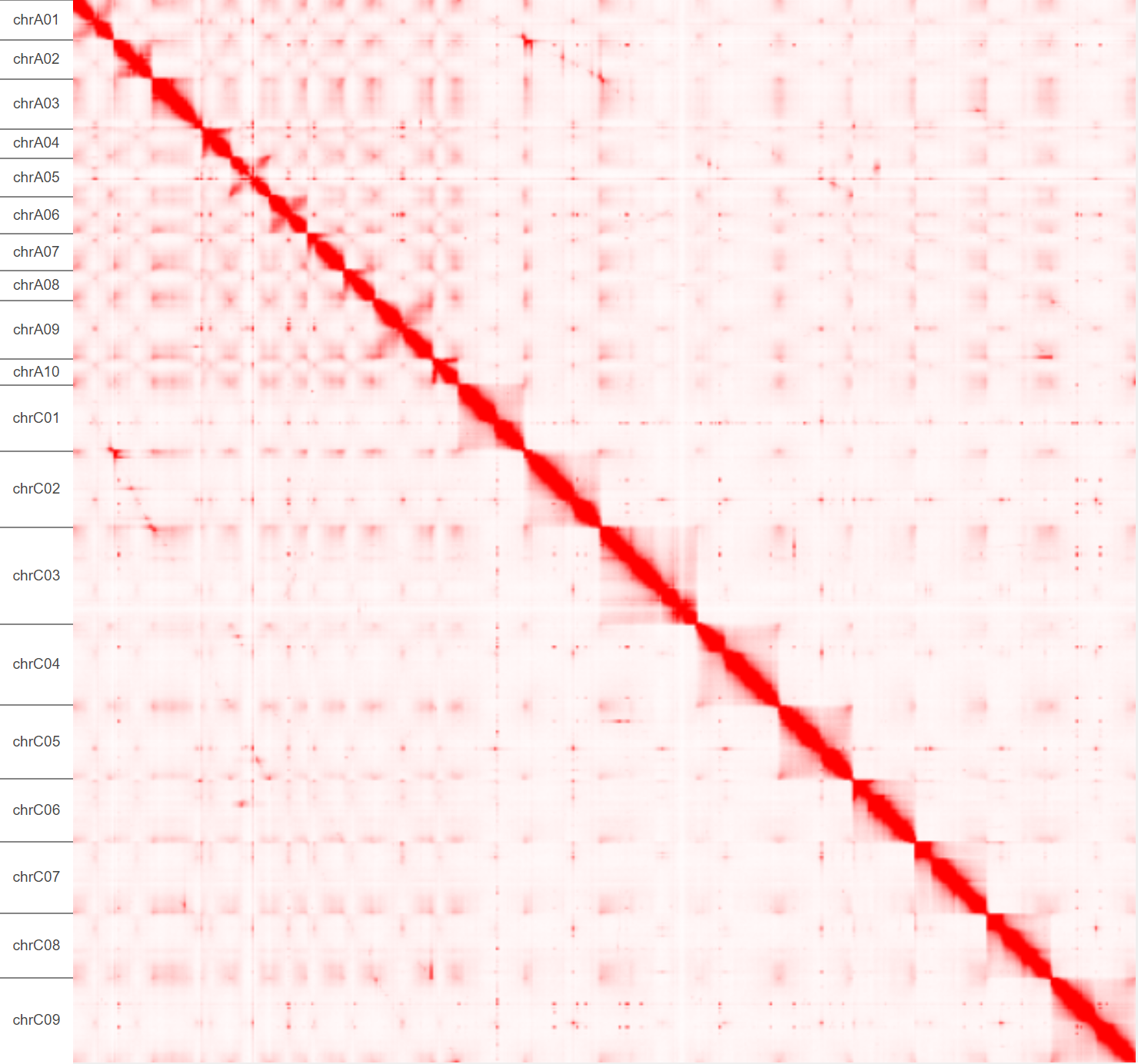


**Figure S2.** Hi-C Contact map.

**
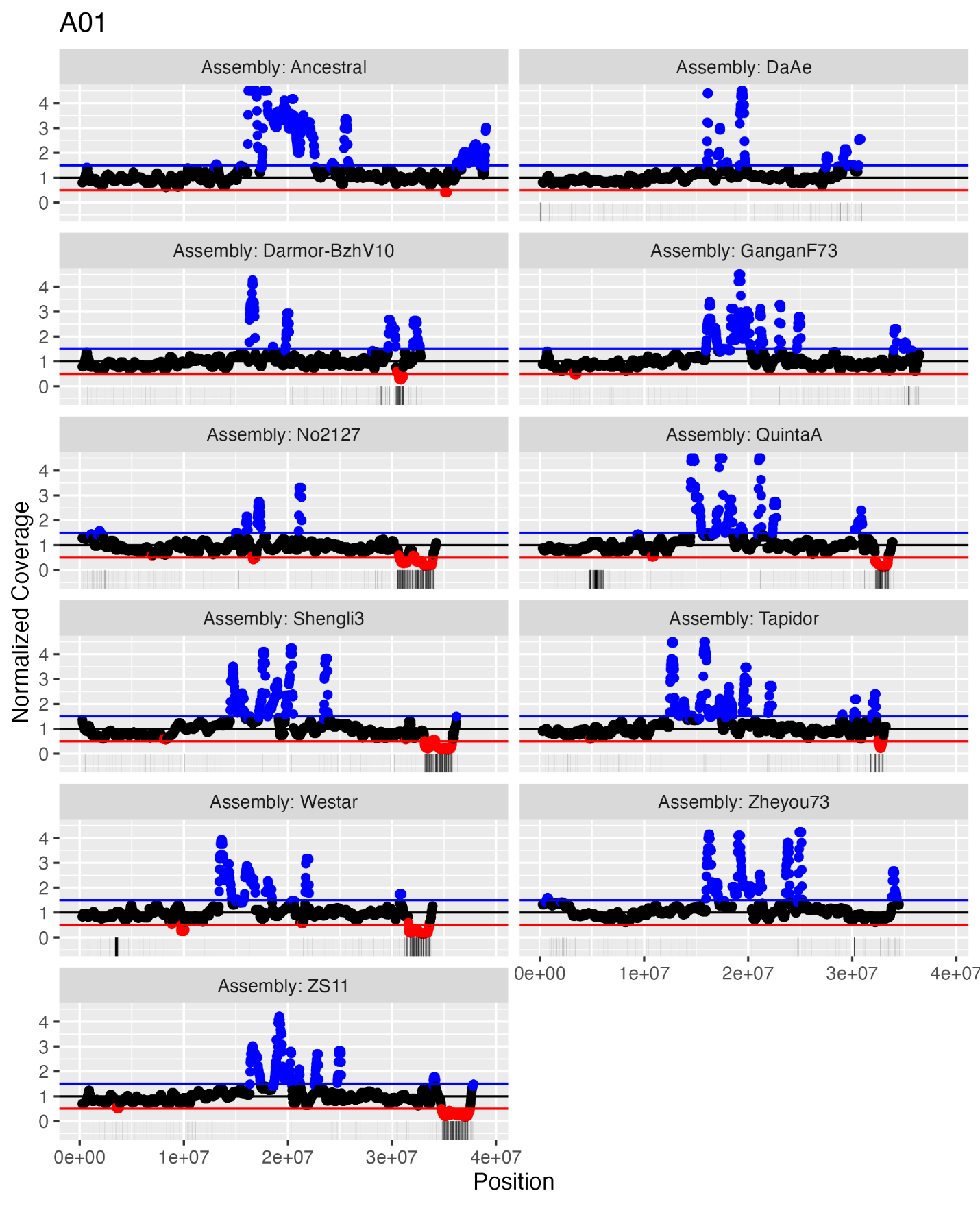
Supplementary Figure S3.**  Coverage of Da-Ae reads mapped to each genome, normalized to the genome-wide median. Areas with coverage less than 0.6x are colored red and those greater than 1.4x are colored blue. Horizontal red, black, and blue lines indicate 0.5x, 1.0x, and 1.5x coverage. Vertical lines below 0 indicate regions of potential homoeologous exchanges based on synteny analysis. Max coverages is capped at 4.5x for readability.


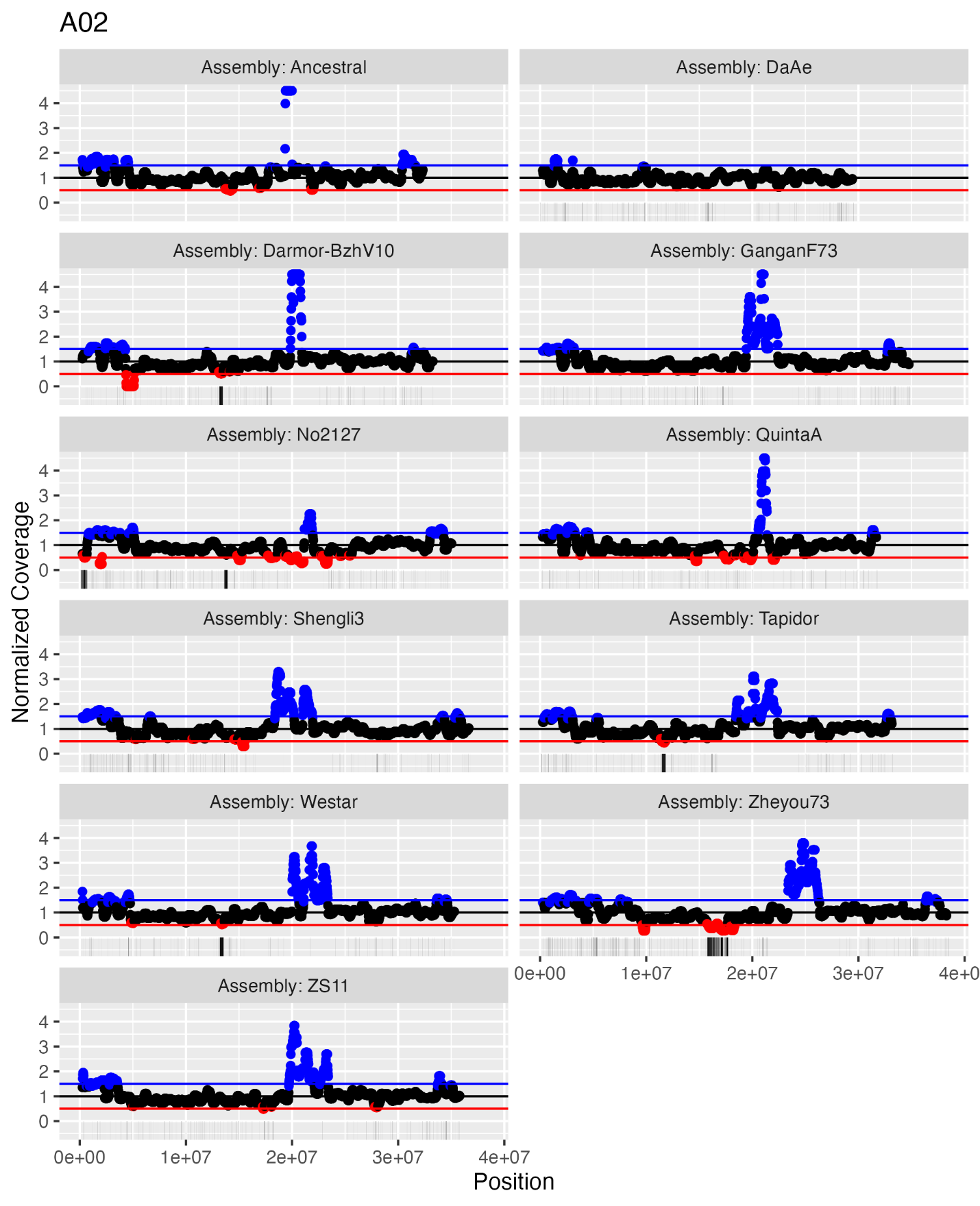
 **Supplementary Figure S4.**  Coverage of Da-Ae reads mapped to each genome, normalized to the genome-wide median. Areas with coverage less than 0.6x are colored red and those greater than 1.4x are colored blue. Horizontal red, black, and blue lines indicate 0.5x, 1.0x, and 1.5x coverage. Vertical lines below 0 indicate regions of potential homoeologous exchanges based on synteny analysis. Max coverages is capped at 4.5x for readability.


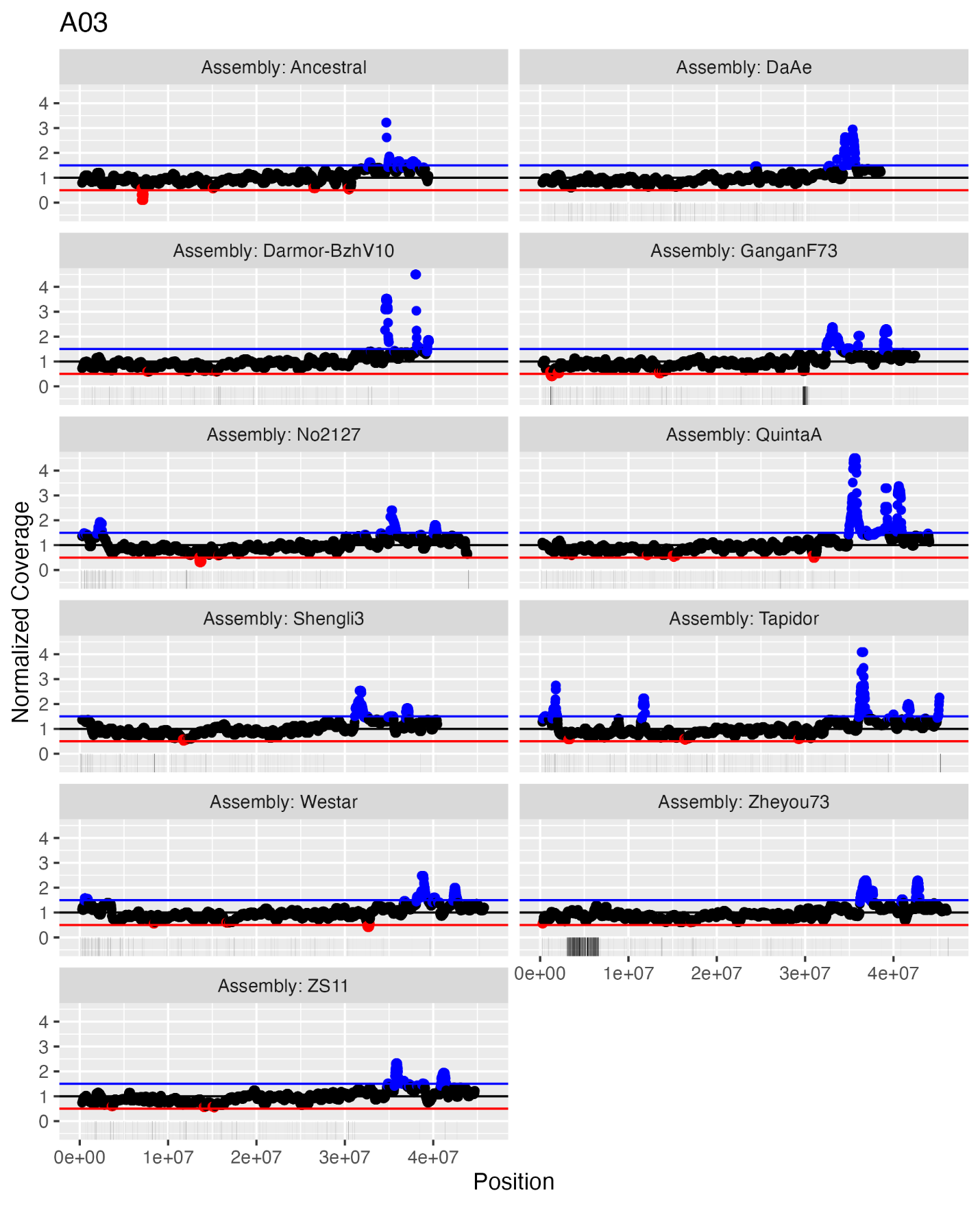
 **Supplementary Figure S5.**  Coverage of Da-Ae reads mapped to each genome, normalized to the genome-wide median. Areas with coverage less than 0.6x are colored red and those greater than 1.4x are colored blue. Horizontal red, black, and blue lines indicate 0.5x, 1.0x, and 1.5x coverage. Vertical lines below 0 indicate regions of potential homoeologous exchanges based on synteny analysis. Max coverages is capped at 4.5x for readability.


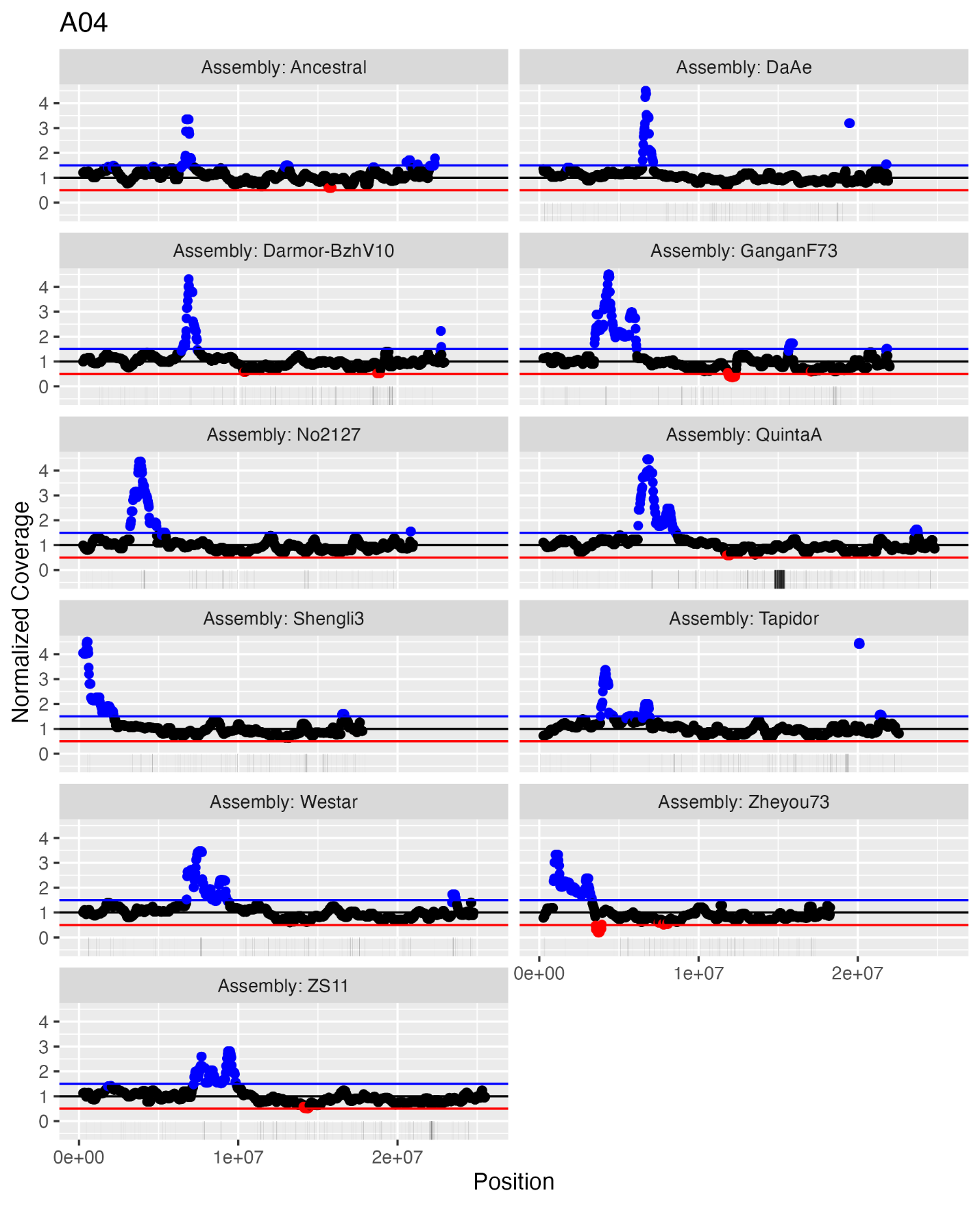
 **Supplementary Figure S6.**  Coverage of Da-Ae reads mapped to each genome, normalized to the genome-wide median. Areas with coverage less than 0.6x are colored red and those greater than 1.4x are colored blue. Horizontal red, black, and blue lines indicate 0.5x, 1.0x, and 1.5x coverage. Vertical lines below 0 indicate regions of potential homoeologous exchanges based on synteny analysis. Max coverages is capped at 4.5x for readability.


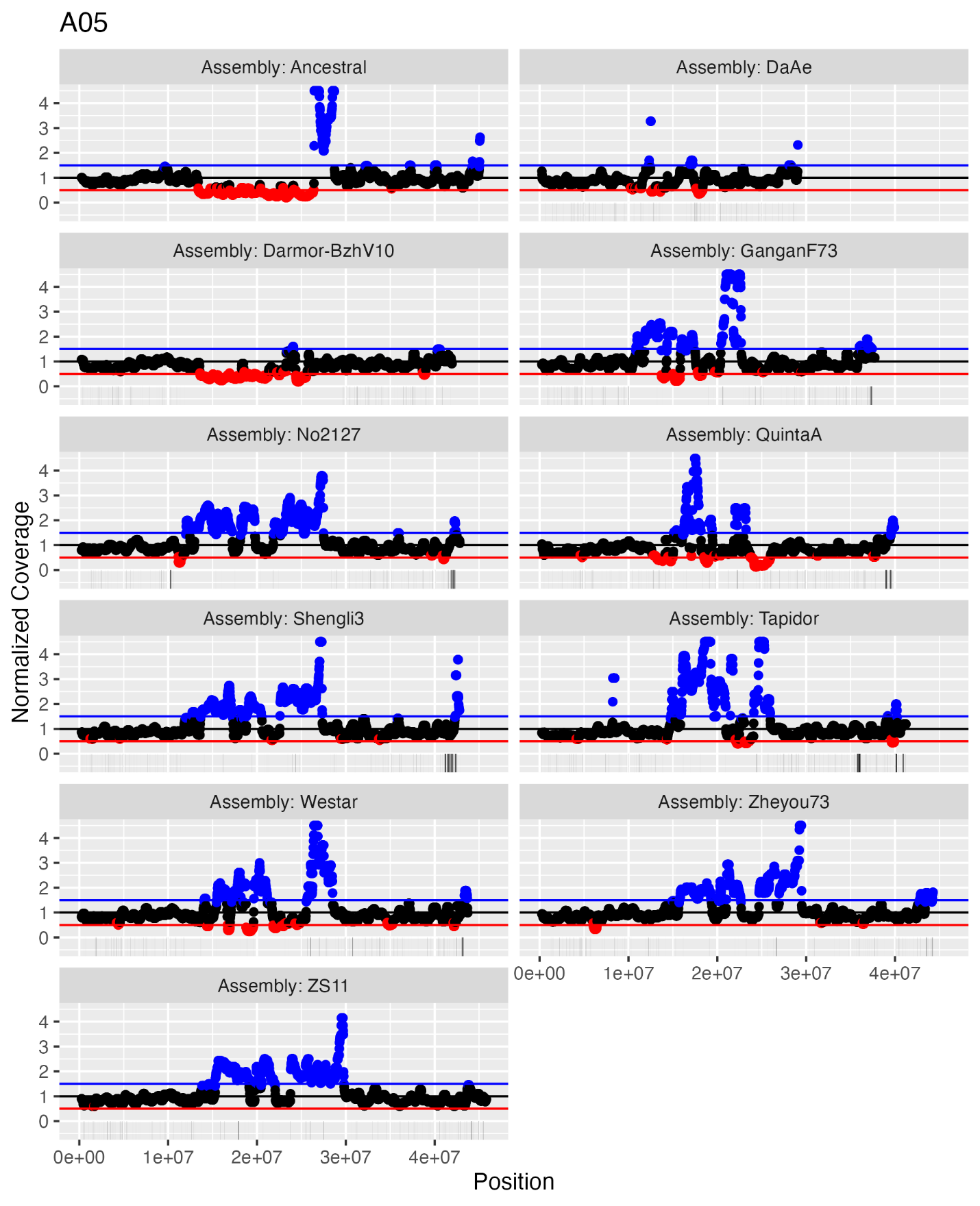
 **Supplementary Figure S7.**  Coverage of Da-Ae reads mapped to each genome, normalized to the genome-wide median. Areas with coverage less than 0.6x are colored red and those greater than 1.4x are colored blue. Horizontal red, black, and blue lines indicate 0.5x, 1.0x, and 1.5x coverage. Vertical lines below 0 indicate regions of potential homoeologous exchanges based on synteny analysis. Max coverages is capped at 4.5x for readability.


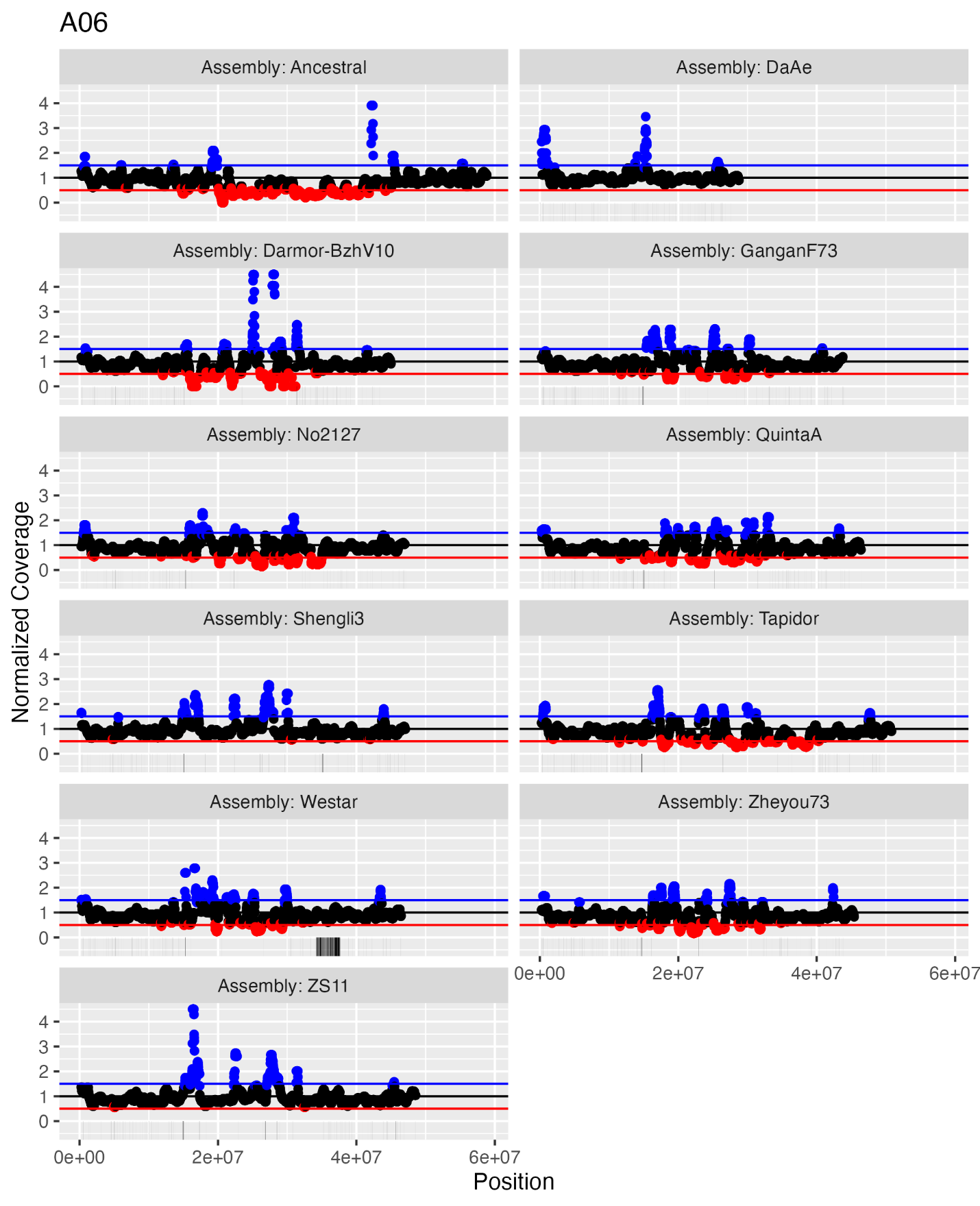
 **Supplementary Figure S8.**  Coverage of Da-Ae reads mapped to each genome, normalized to the genome-wide median. Areas with coverage less than 0.6x are colored red and those greater than 1.4x are colored blue. Horizontal red, black, and blue lines indicate 0.5x, 1.0x, and 1.5x coverage. Vertical lines below 0 indicate regions of potential homoeologous exchanges based on synteny analysis. Max coverages is capped at 4.5x for readability.


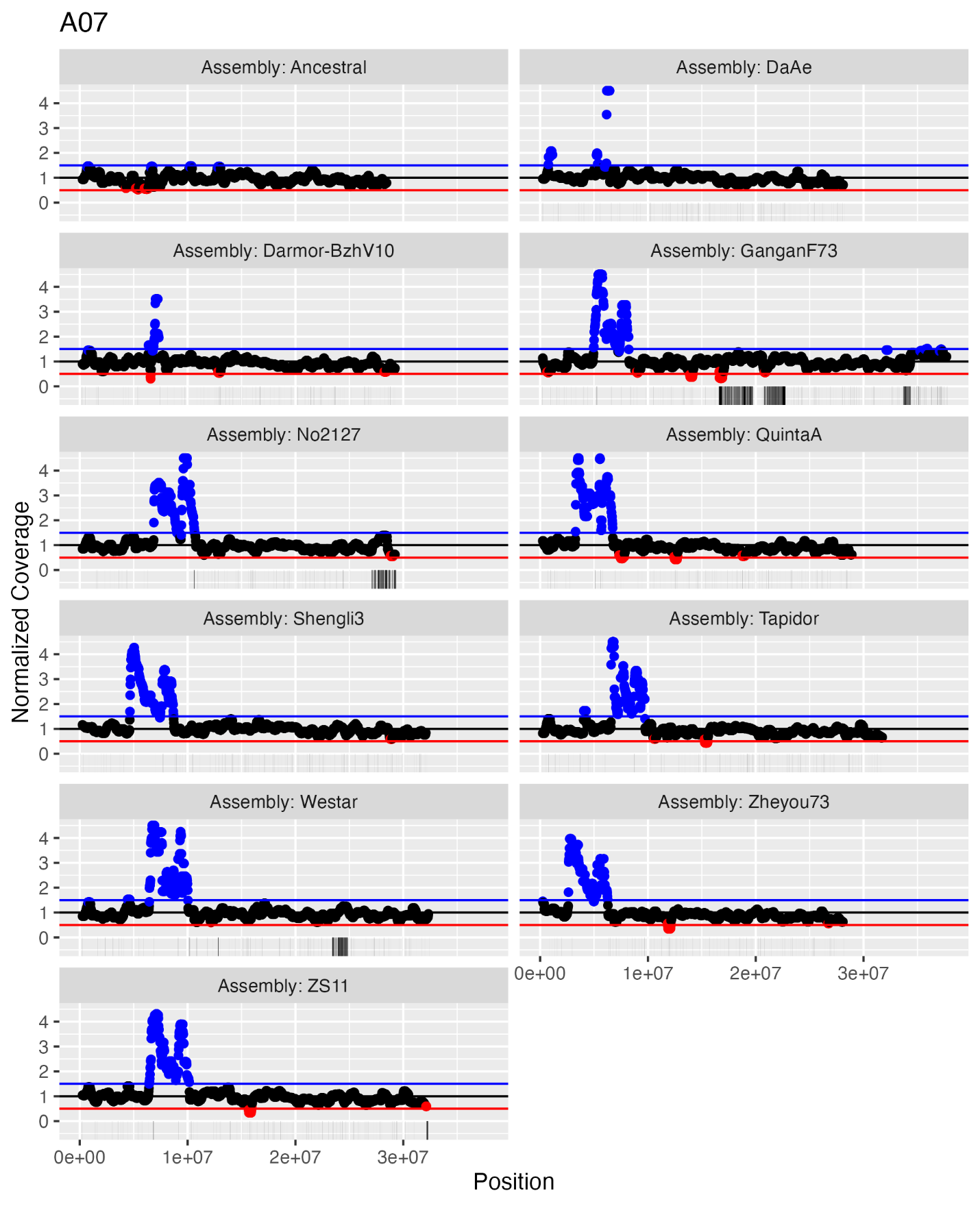
 **Supplementary Figure S9.**  Coverage of Da-Ae reads mapped to each genome, normalized to the genome-wide median. Areas with coverage less than 0.6x are colored red and those greater than 1.4x are colored blue. Horizontal red, black, and blue lines indicate 0.5x, 1.0x, and 1.5x coverage. Vertical lines below 0 indicate regions of potential homoeologous exchanges based on synteny analysis. Max coverages is capped at 4.5x for readability.


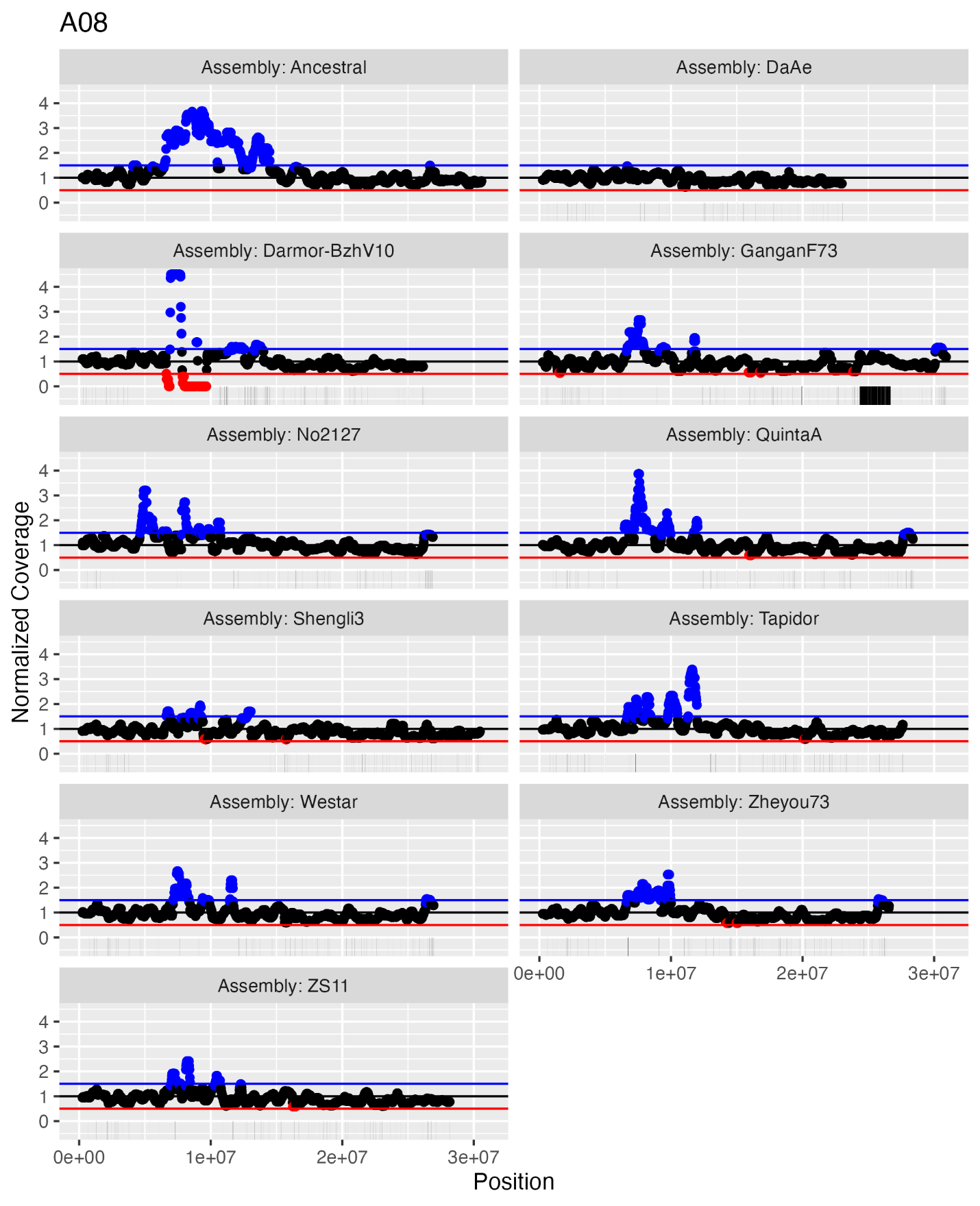
 **Supplementary Figure S10.**  Coverage of Da-Ae reads mapped to each genome, normalized to the genome-wide median. Areas with coverage less than 0.6x are colored red and those greater than 1.4x are colored blue. Horizontal red, black, and blue lines indicate 0.5x, 1.0x, and 1.5x coverage. Vertical lines below 0 indicate regions of potential homoeologous exchanges based on synteny analysis. Max coverages is capped at 4.5x for readability.


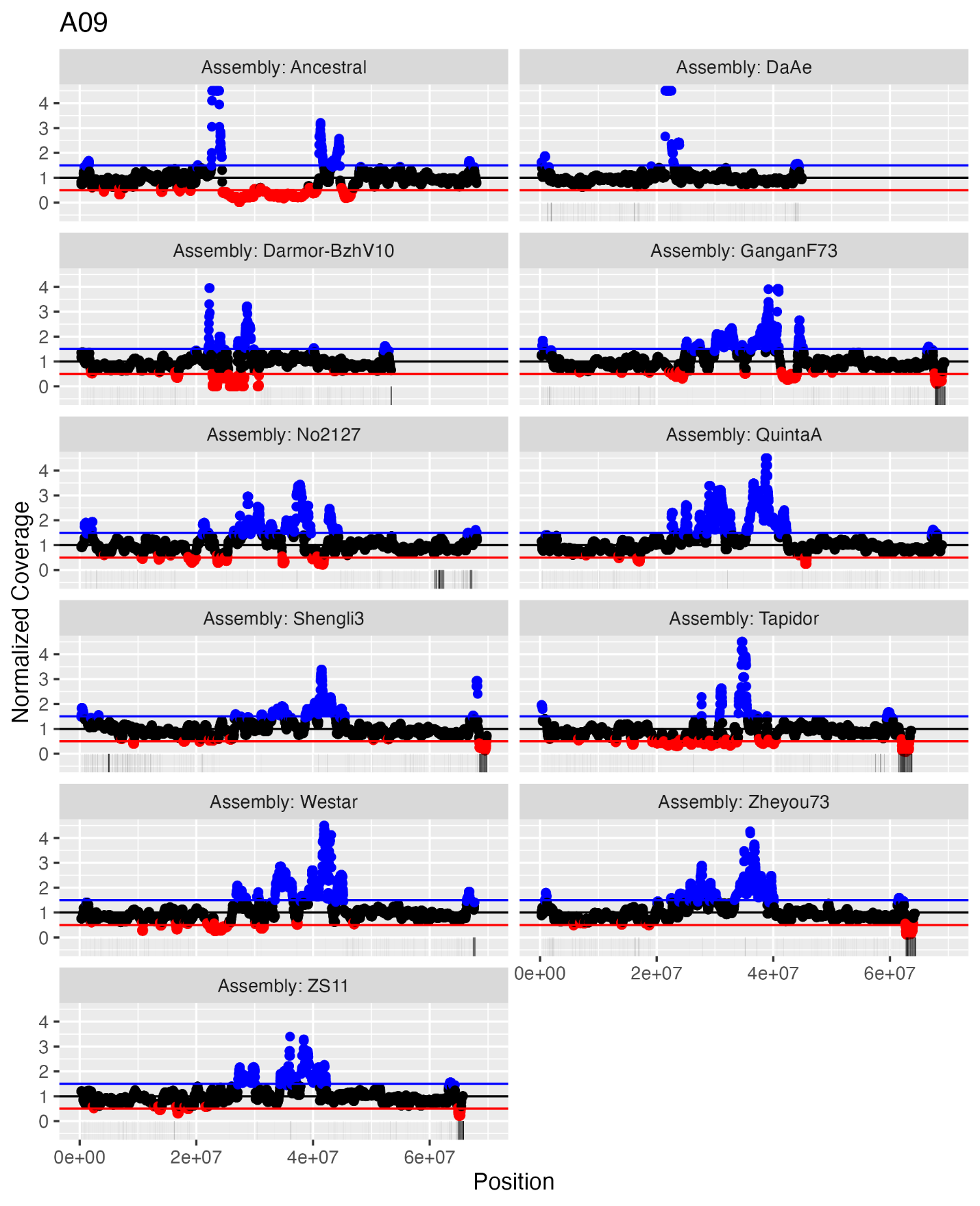
 **Supplementary Figure S11.** Coverage of Da-Ae reads mapped to each genome, normalized to the genome-wide median. Areas with coverage less than 0.6x are colored red and those greater than 1.4x are colored blue. Horizontal red, black, and blue lines indicate 0.5x, 1.0x, and 1.5x coverage. Vertical lines below 0 indicate regions of potential homoeologous exchanges based on synteny analysis. Max coverages is capped at 4.5x for readability.


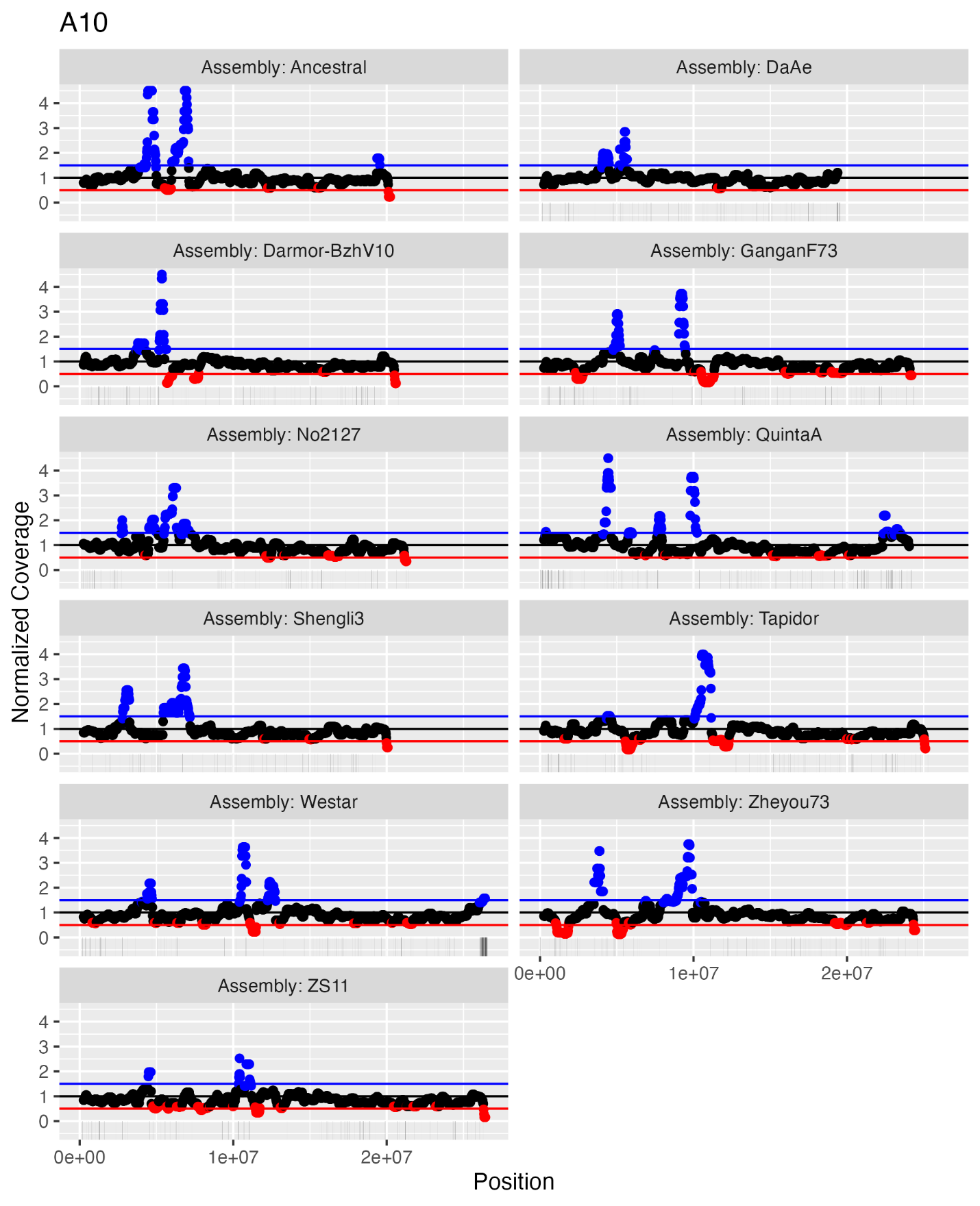
 **Supplementary Figure S12.** Coverage of Da-Ae reads mapped to each genome, normalized to the genome-wide median. Areas with coverage less than 0.6x are colored red and those greater than 1.4x are colored blue. Horizontal red, black, and blue lines indicate 0.5x, 1.0x, and 1.5x coverage. Vertical lines below 0 indicate regions of potential homoeologous exchanges based on synteny analysis. Max coverages is capped at 4.5x for readability.


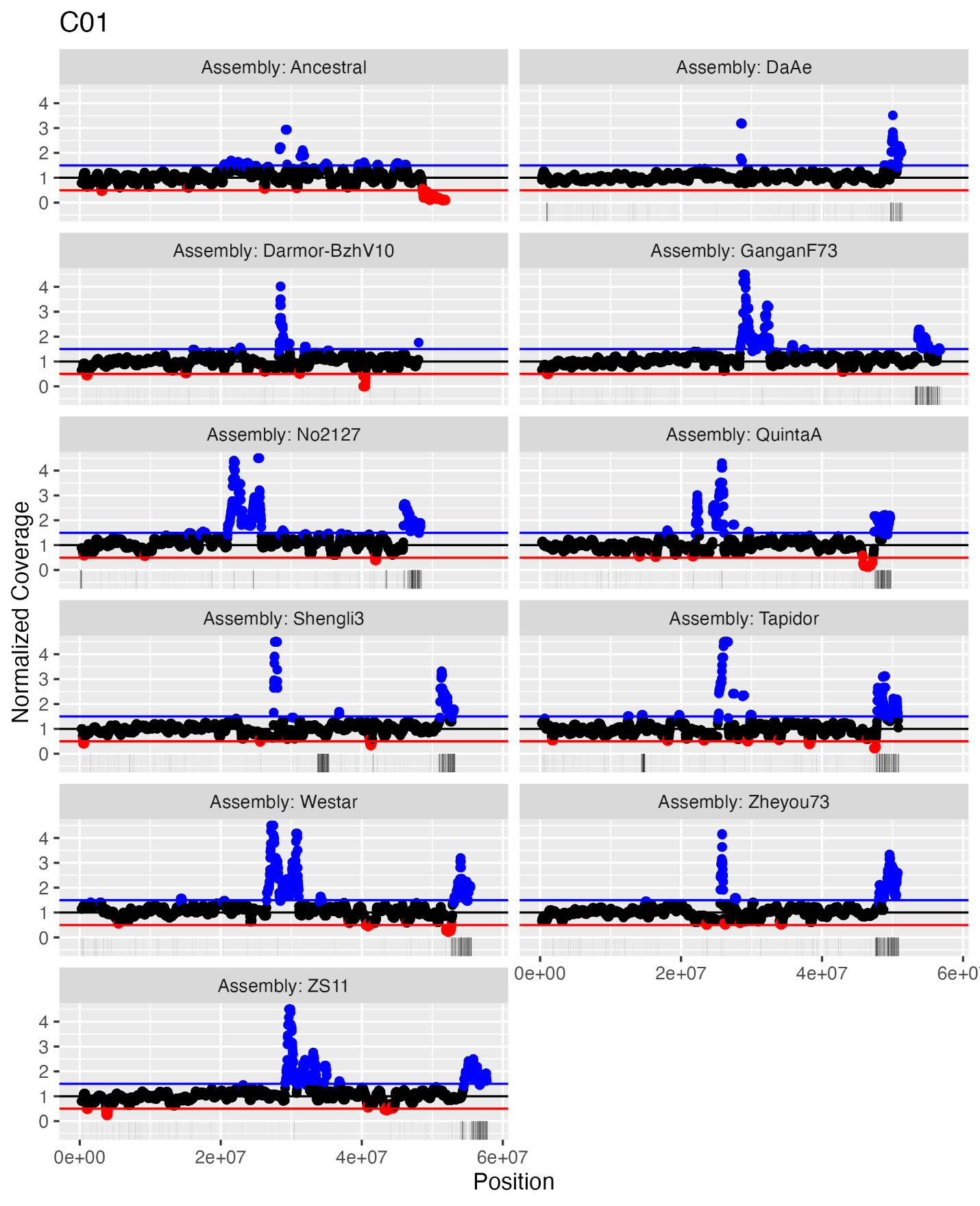
 **Supplementary Figure S13.**  Coverage of Da-Ae reads mapped to each genome, normalized to the genome-wide median. Areas with coverage less than 0.6x are colored red and those greater than 1.4x are colored blue. Horizontal red, black, and blue lines indicate 0.5x, 1.0x, and 1.5x coverage. Vertical lines below 0 indicate regions of potential homoeologous exchanges based on synteny analysis. Max coverages is capped at 4.5x for readability.


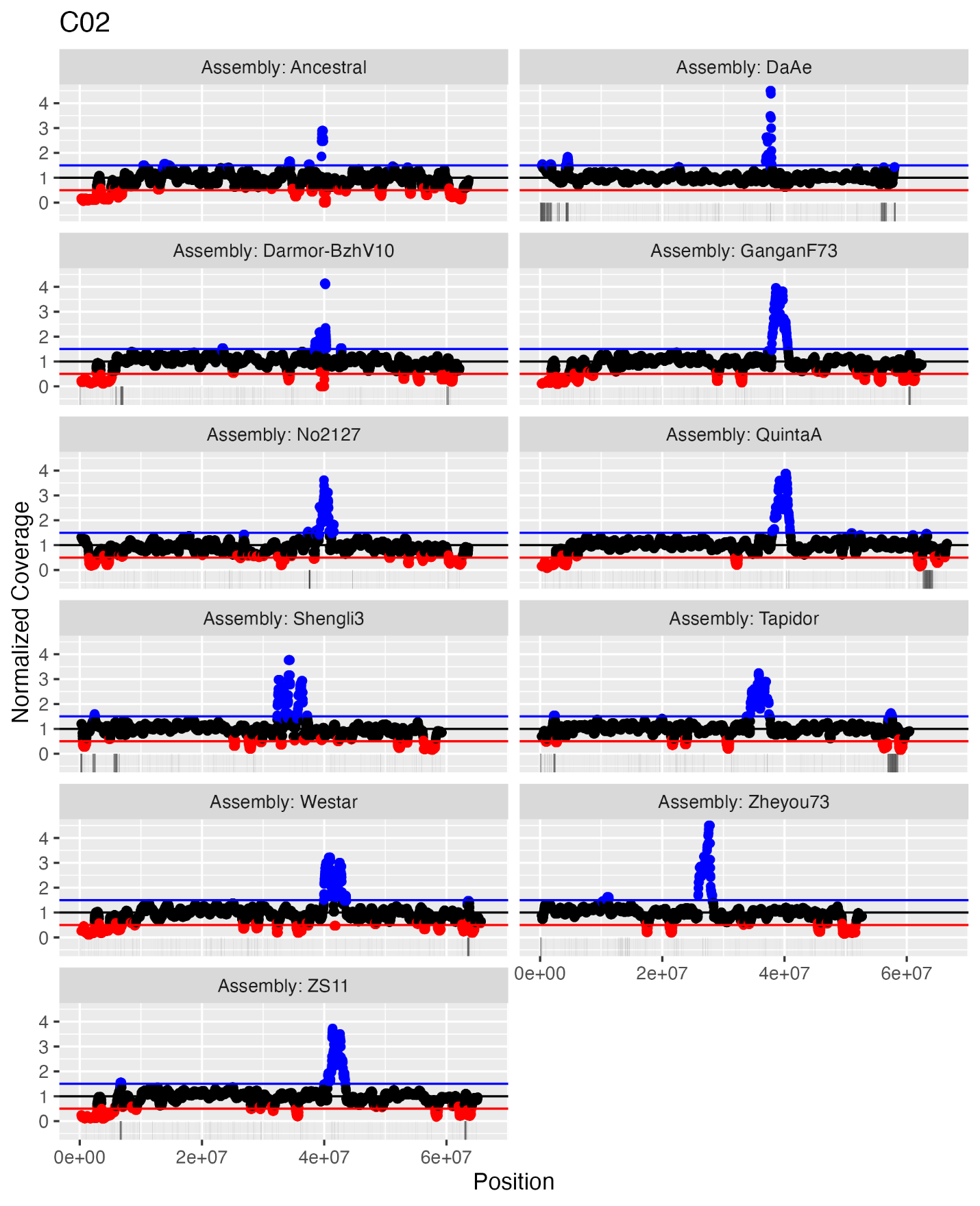
 **Supplementary Figure S14.**  Coverage of Da-Ae reads mapped to each genome, normalized to the genome-wide median. Areas with coverage less than 0.6x are colored red and those greater than 1.4x are colored blue. Horizontal red, black, and blue lines indicate 0.5x, 1.0x, and 1.5x coverage. Vertical lines below 0 indicate regions of potential homoeologous exchanges based on synteny analysis. Max coverages is capped at 4.5x for readability.


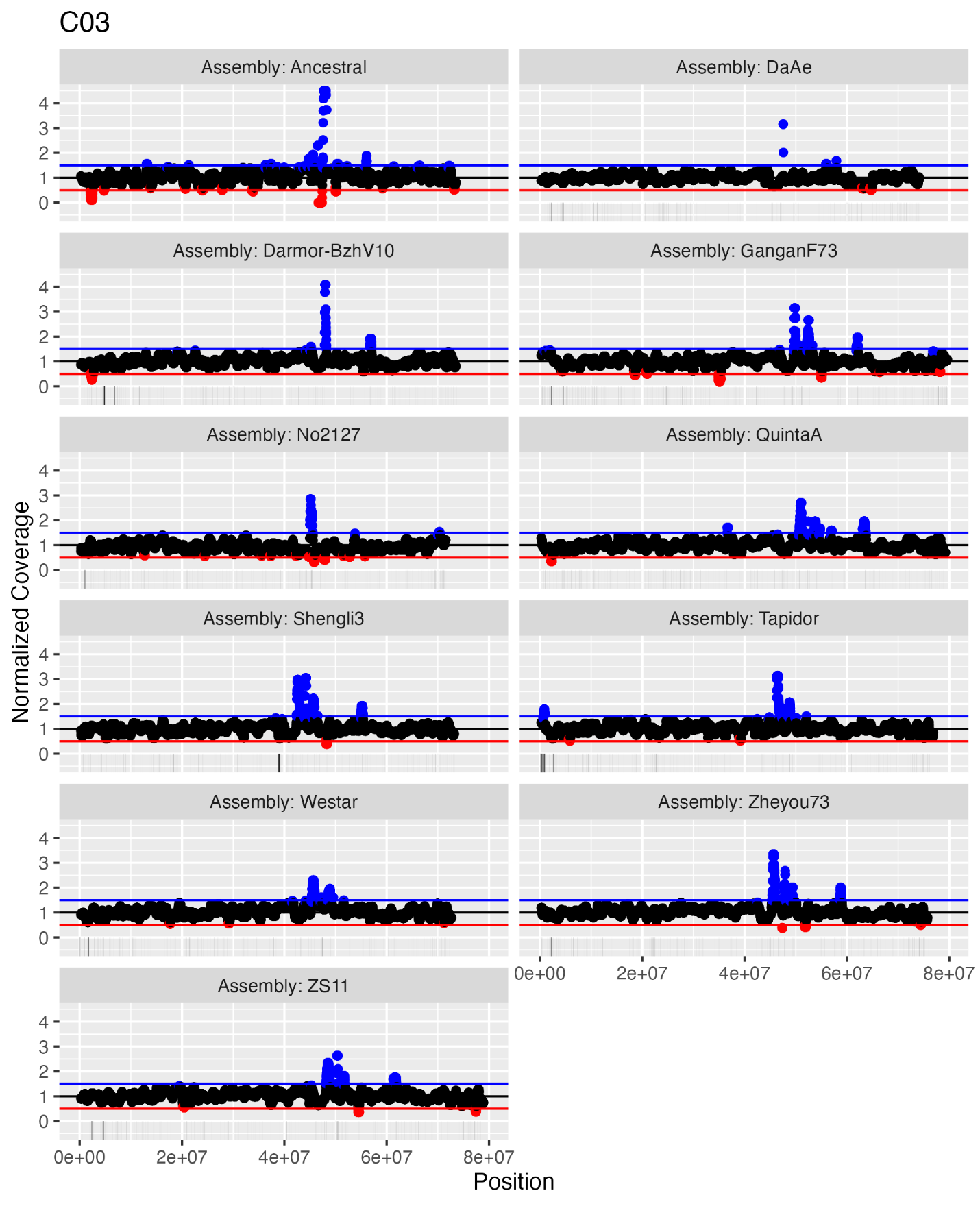
 **Supplementary Figure S15.**  Coverage of Da-Ae reads mapped to each genome, normalized to the genome-wide median. Areas with coverage less than 0.6x are colored red and those greater than 1.4x are colored blue. Horizontal red, black, and blue lines indicate 0.5x, 1.0x, and 1.5x coverage. Vertical lines below 0 indicate regions of potential homoeologous exchanges based on synteny analysis. Max coverages is capped at 4.5x for readability.


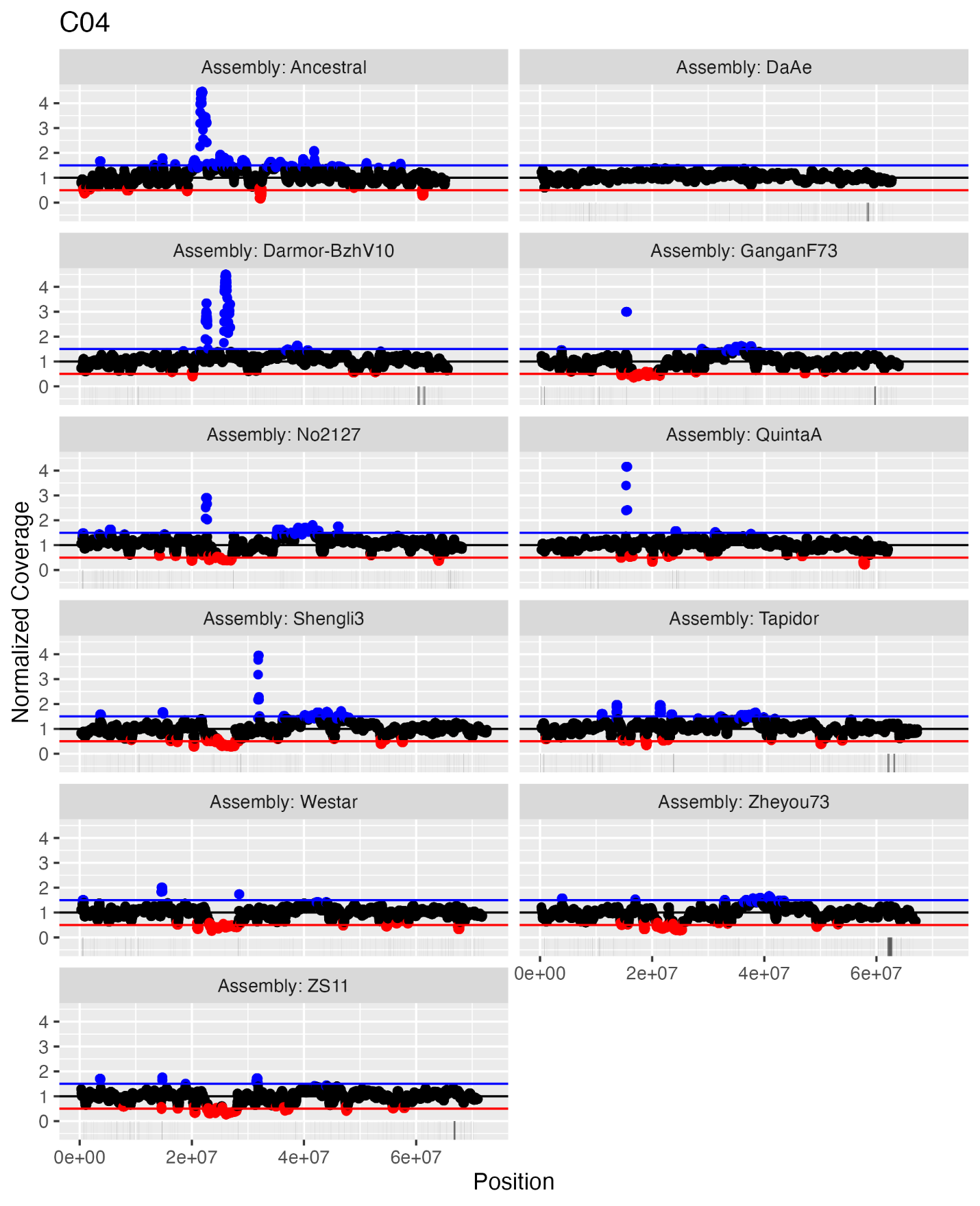
 **Supplementary Figure S16.**  Coverage of Da-Ae reads mapped to each genome, normalized to the genome-wide median. Areas with coverage less than 0.6x are colored red and those greater than 1.4x are colored blue. Horizontal red, black, and blue lines indicate 0.5x, 1.0x, and 1.5x coverage. Vertical lines below 0 indicate regions of potential homoeologous exchanges based on synteny analysis. Max coverages is capped at 4.5x for readability.


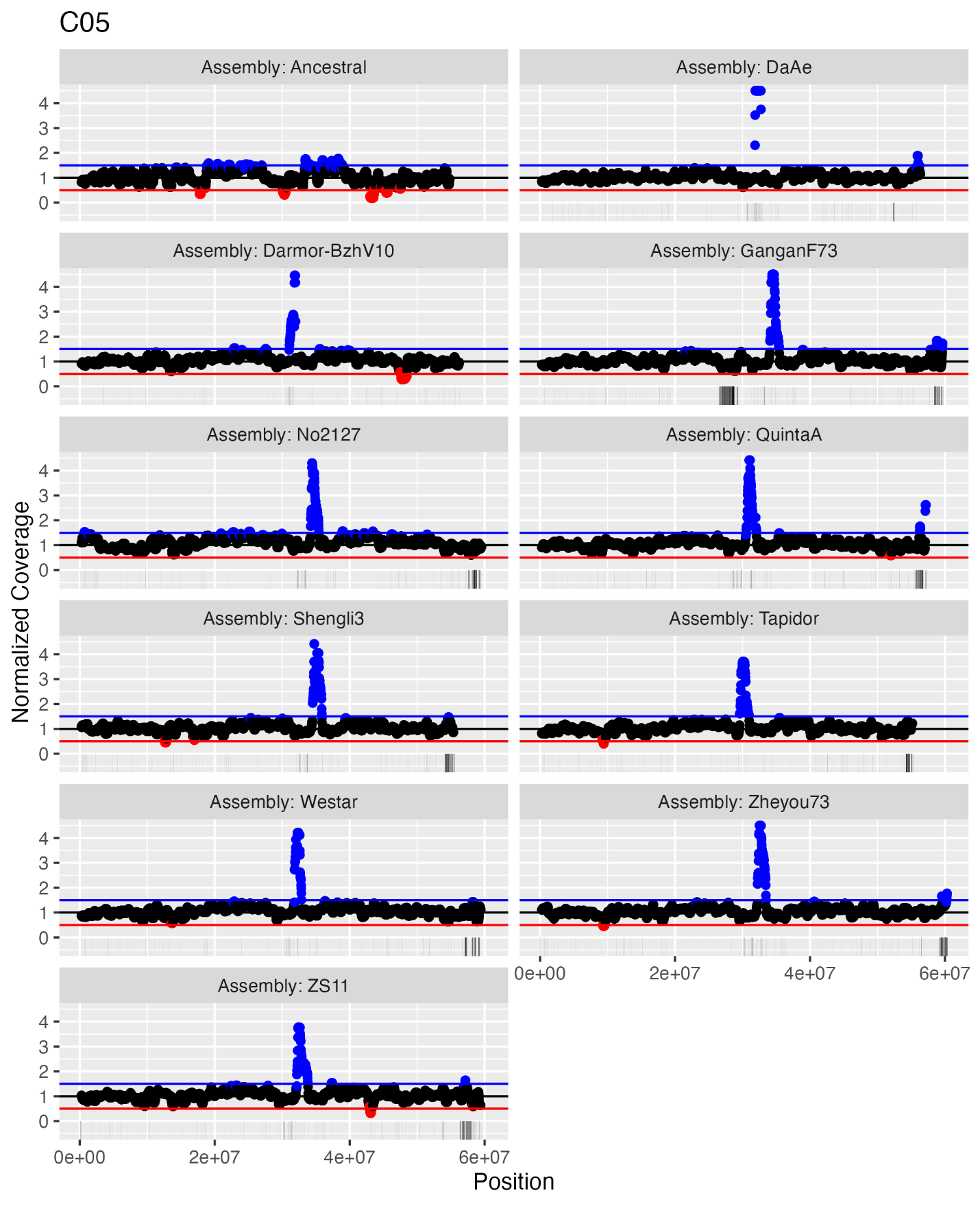
 **Supplementary Figure S17.**  Coverage of Da-Ae reads mapped to each genome, normalized to the genome-wide median. Areas with coverage less than 0.6x are colored red and those greater than 1.4x are colored blue. Horizontal red, black, and blue lines indicate 0.5x, 1.0x, and 1.5x coverage. Vertical lines below 0 indicate regions of potential homoeologous exchanges based on synteny analysis. Max coverages is capped at 4.5x for readability.


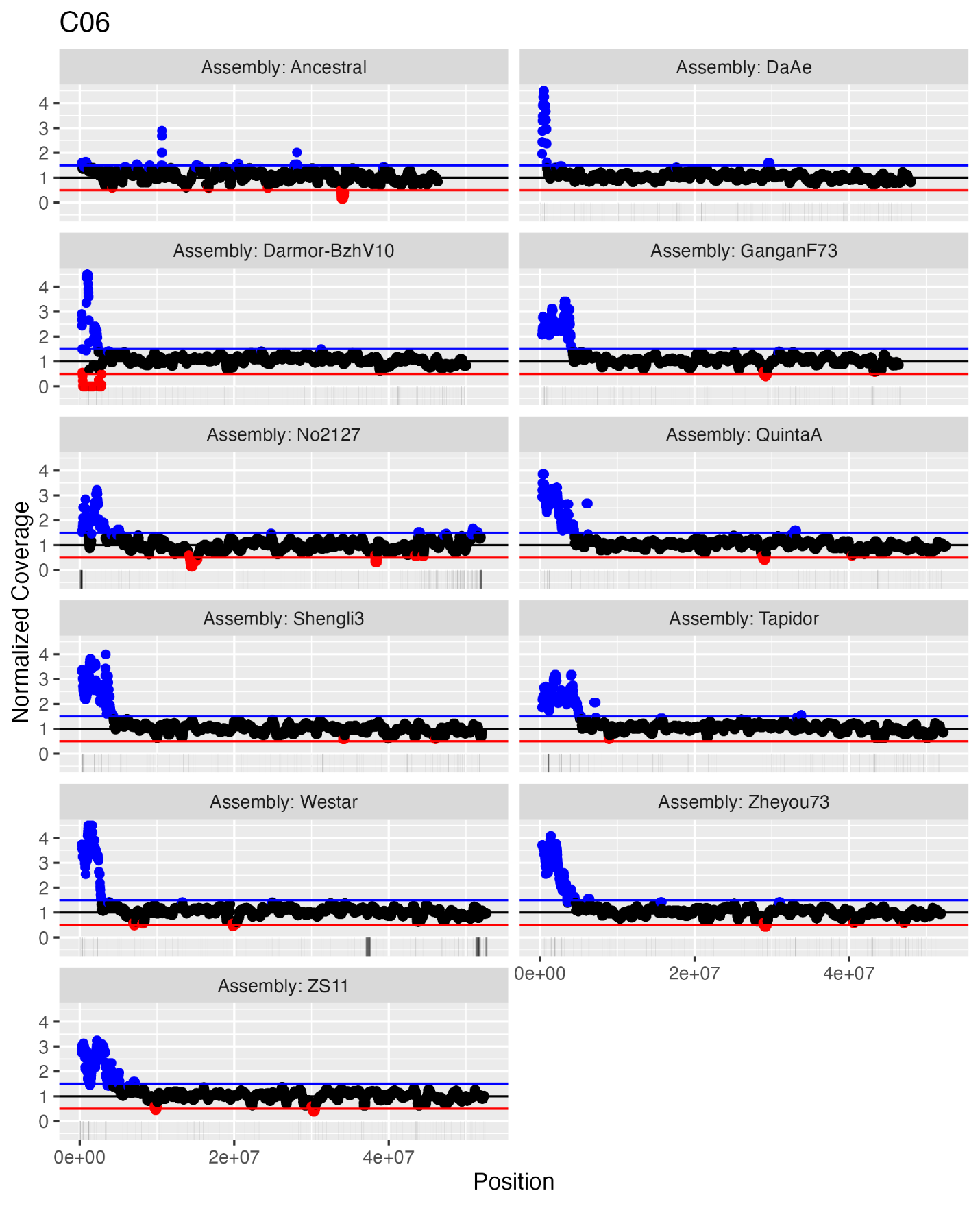
 **Supplementary Figure S18.**  Coverage of Da-Ae reads mapped to each genome, normalized to the genome-wide median. Areas with coverage less than 0.6x are colored red and those greater than 1.4x are colored blue. Horizontal red, black, and blue lines indicate 0.5x, 1.0x, and 1.5x coverage. Vertical lines below 0 indicate regions of potential homoeologous exchanges based on synteny analysis. Max coverages is capped at 4.5x for readability.


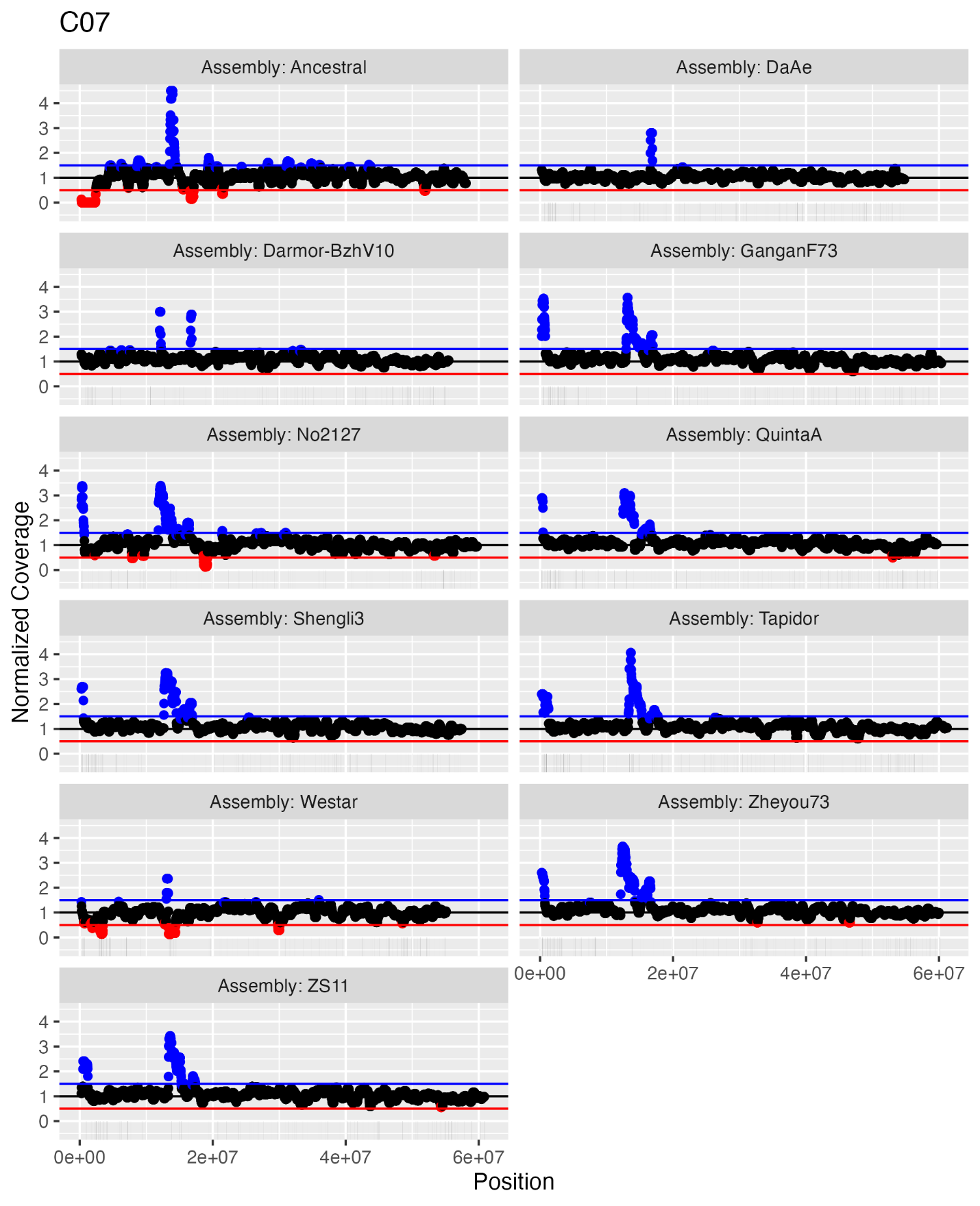
 **Supplementary Figure S19.**  Coverage of Da-Ae reads mapped to each genome, normalized to the genome-wide median. Areas with coverage less than 0.6x are colored red and those greater than 1.4x are colored blue. Horizontal red, black, and blue lines indicate 0.5x, 1.0x, and 1.5x coverage. Vertical lines below 0 indicate regions of potential homoeologous exchanges based on synteny analysis. Max coverages is capped at 4.5x for readability.


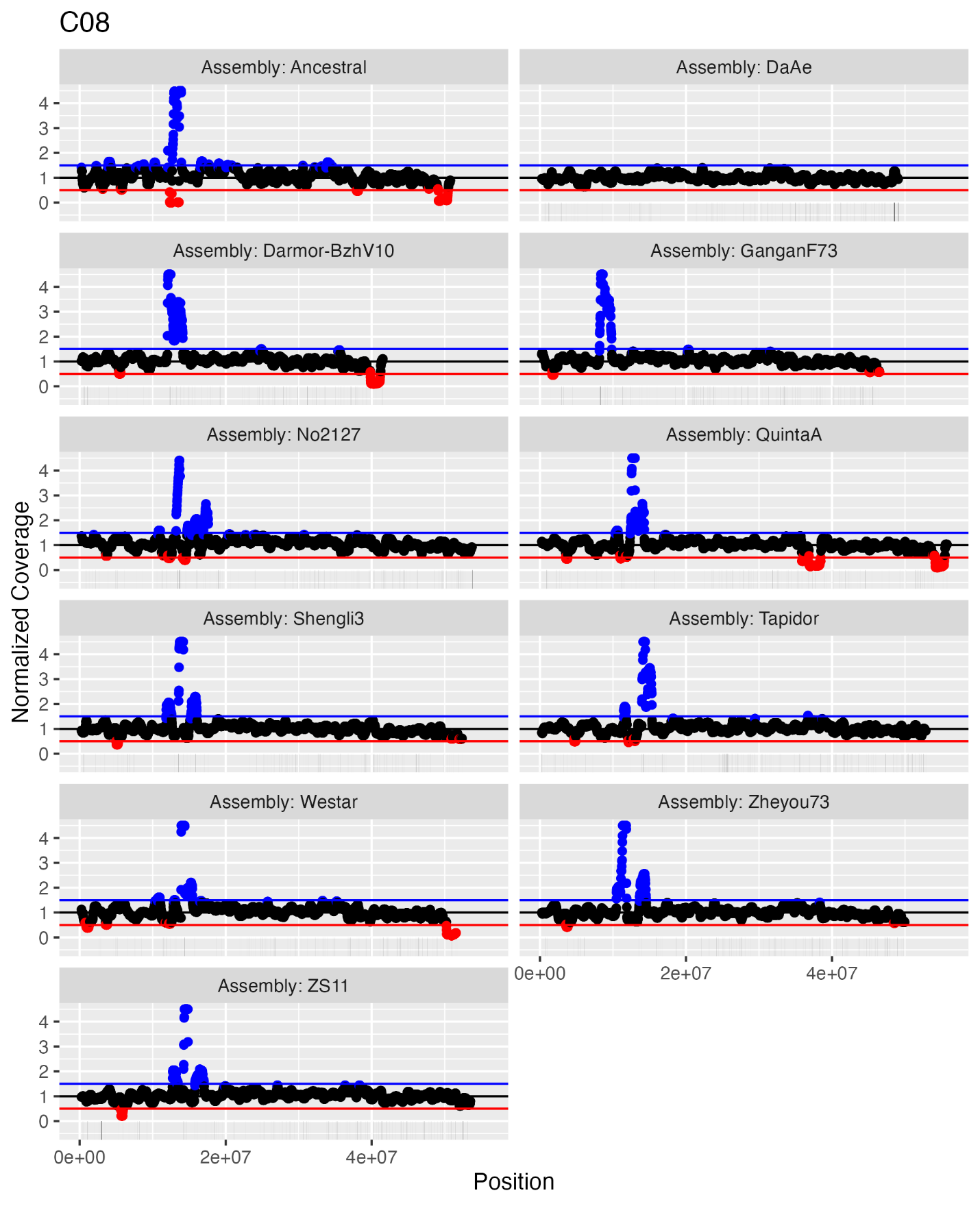
 **Supplementary Figure S20.**  Coverage of Da-Ae reads mapped to each genome, normalized to the genome-wide median. Areas with coverage less than 0.6x are colored red and those greater than 1.4x are colored blue. Horizontal red, black, and blue lines indicate 0.5x, 1.0x, and 1.5x coverage. Vertical lines below 0 indicate regions of potential homoeologous exchanges based on synteny analysis. Max coverages is capped at 4.5x for readability.


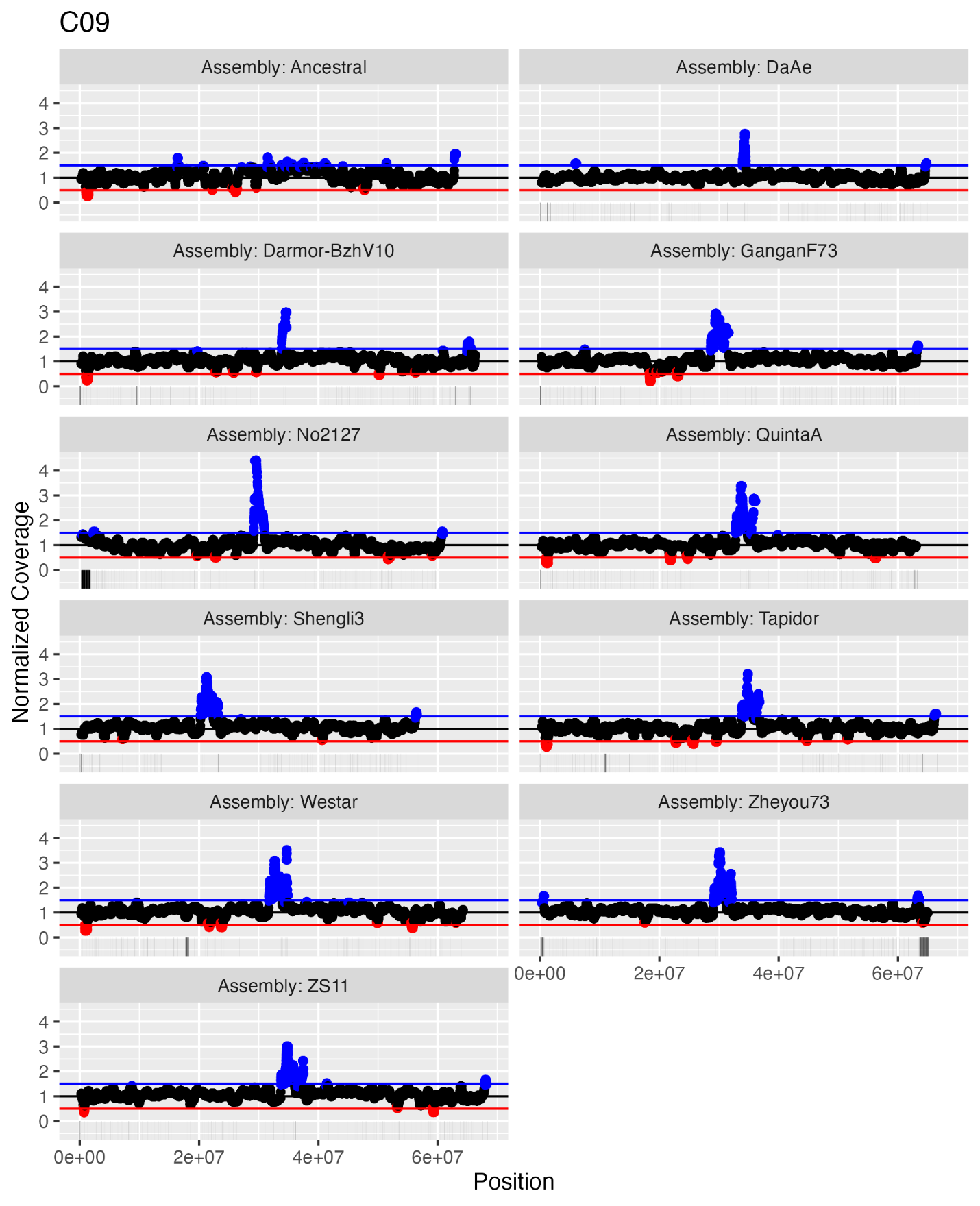
 **Supplementary Figure S21.**  Coverage of Da-Ae reads mapped to each genome, normalized to the genome-wide median. Areas with coverage less than 0.6x are colored red and those greater than 1.4x are colored blue. Horizontal red, black, and blue lines indicate 0.5x, 1.0x, and 1.5x coverage. Vertical lines below 0 indicate regions of potential homoeologous exchanges based on synteny analysis. Max coverages is capped at 4.5x for readability


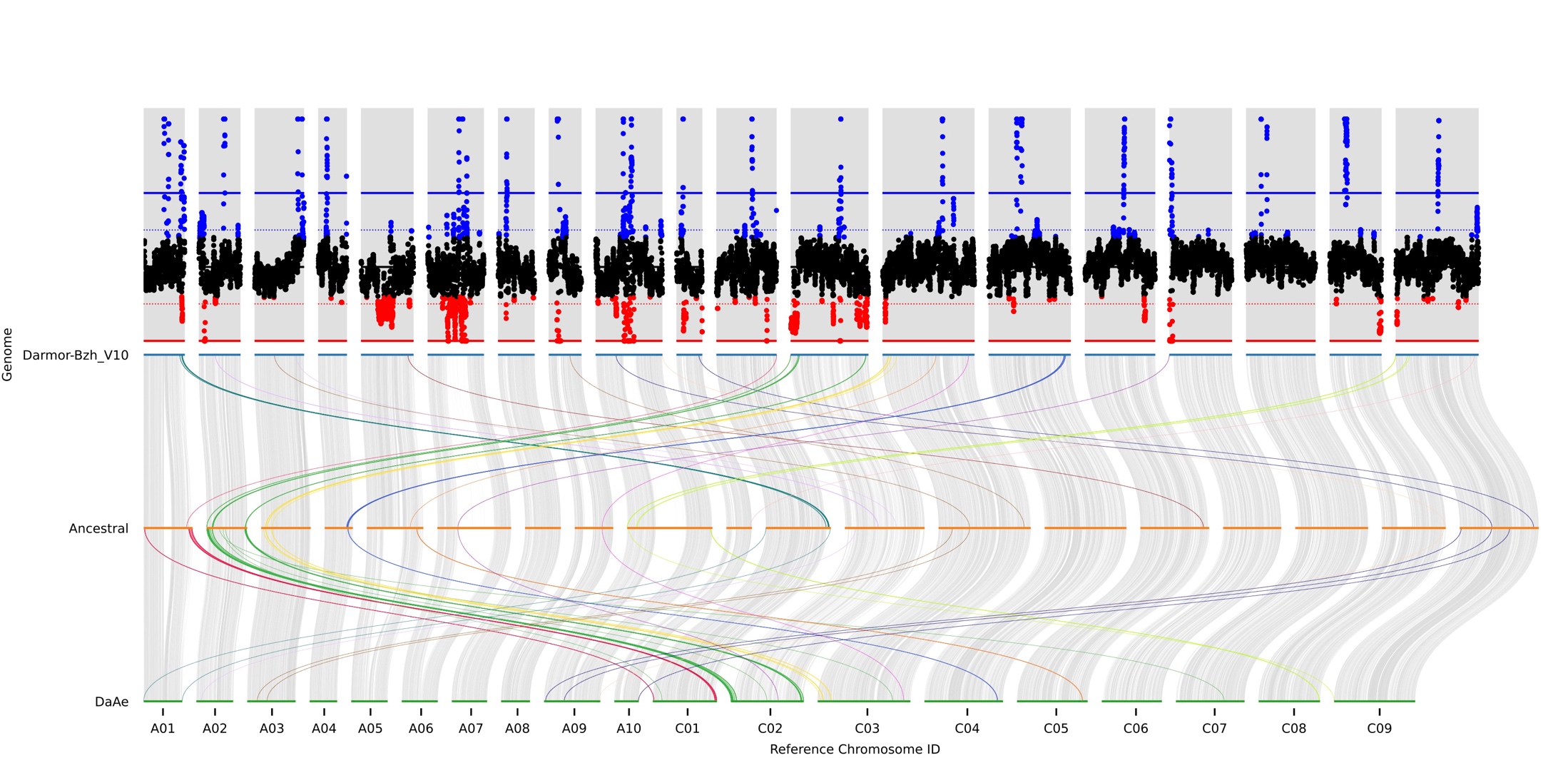


**Figure S22.** Coverage and homoeologous exchange plot. Top panel: Coverage of Da-Ae reads mapped to Darmor Bzh V10; replotted from figures S3 – S21. Vertical lines indicate 0, 0.5x, 1x, 1.5x, and 2x coverage. Bottom panel: homoeologous exchange. Grey lines show homologous regions between the ancestral chromosomes and the two *B. napus* varieties. Colored lines indicate homoeologous exchange; the color of the line corresponds to the ancestral chromosome.


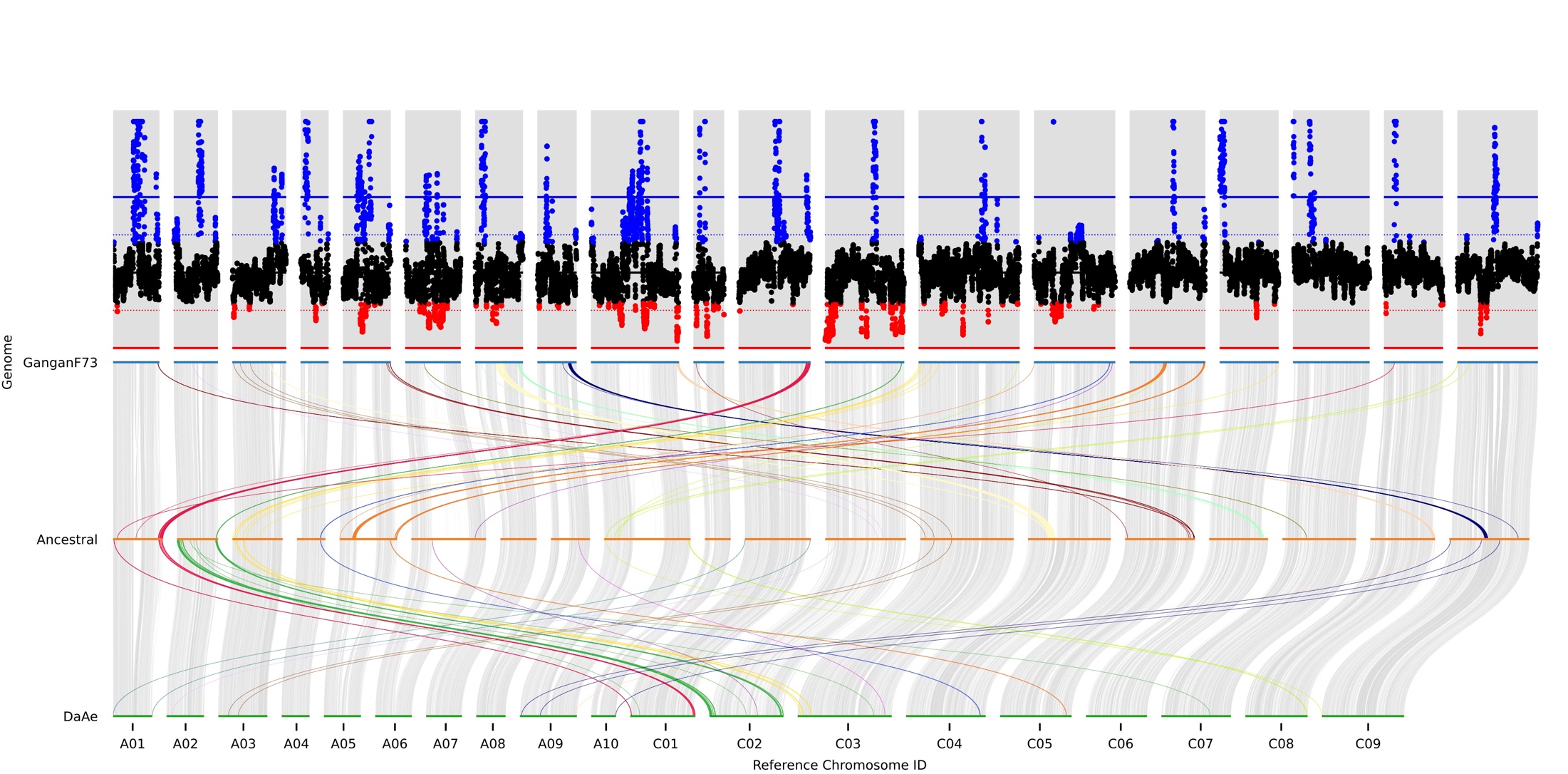


**Figure S23.** Coverage and homoeologous exchange plot. Top panel: Coverage of Da-Ae reads mapped to GanganF73; replotted from figures S3 – S21. Vertical lines indicate 0, 0.5x, 1x, 1.5x, and 2x coverage. Bottom panel: homoeologous exchange. Grey lines show homologous regions between the ancestral chromosomes and the two *B. napus* varieties. Colored lines indicate homoeologous exchange; the color of the line corresponds to the ancestral chromosome.


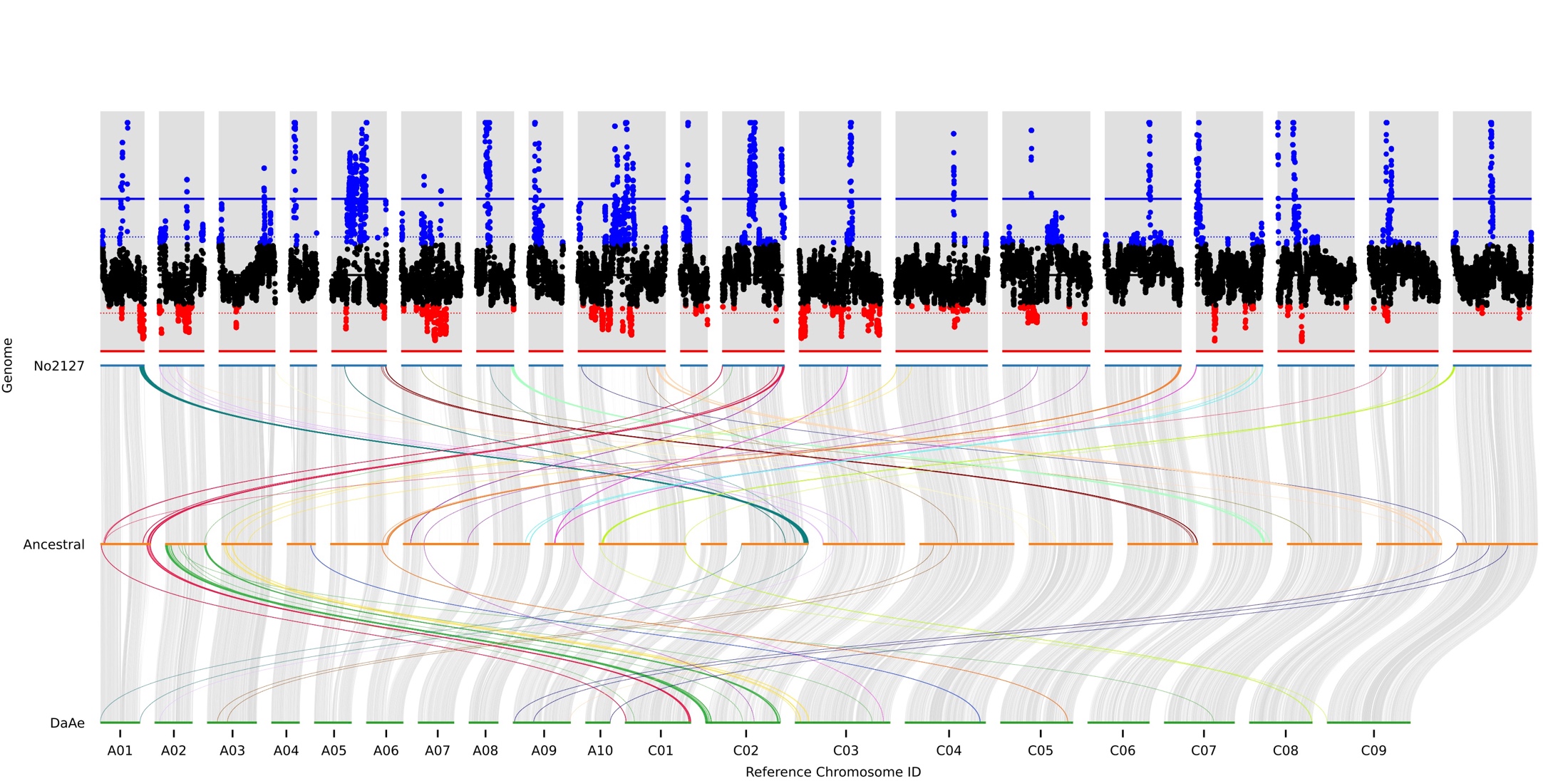


**Figure S24.** Coverage and homoeologous exchange plot. Top panel: Coverage of Da-Ae reads mapped to No1727; replotted from figures S3 – S21. Vertical lines indicate 0, 0.5x, 1x, 1.5x, and 2x coverage. Bottom panel: homoeologous exchange. Grey lines show homologous regions between the ancestral chromosomes and the two *B. napus* varieties. Colored lines indicate homoeologous exchange; the color of the line corresponds to the ancestral chromosome.


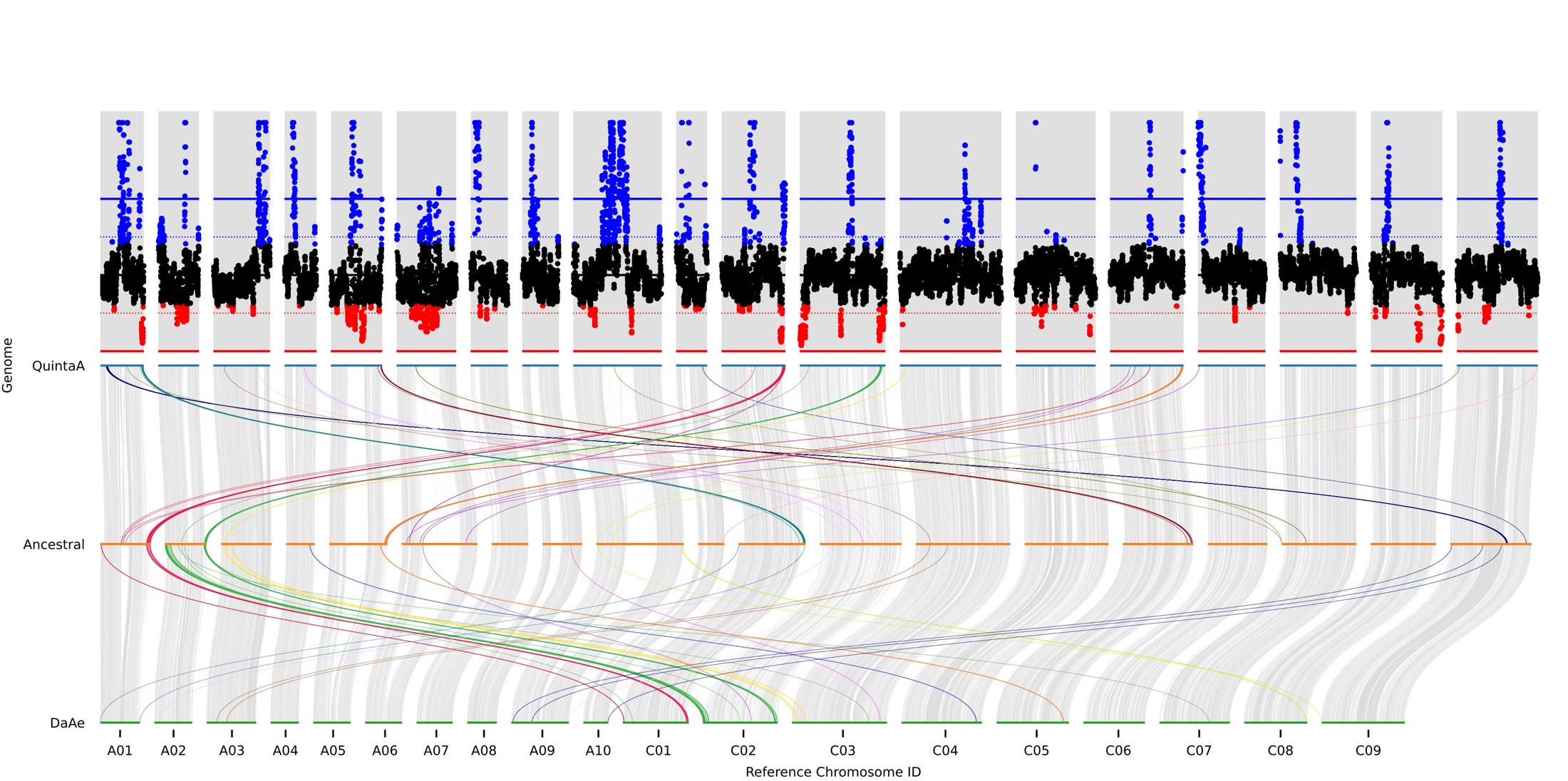


**Figure S25.** Coverage and homoeologous exchange plot. Top panel: Coverage of Da-Ae reads mapped to QuintaA; replotted from figures S3 – S21. Vertical lines indicate 0, 0.5x, 1x, 1.5x, and 2x coverage. Bottom panel: homoeologous exchange. Grey lines show homologous regions between the ancestral chromosomes and the two *B. napus* varieties. Colored lines indicate homoeologous exchange; the color of the line corresponds to the ancestral chromosome.


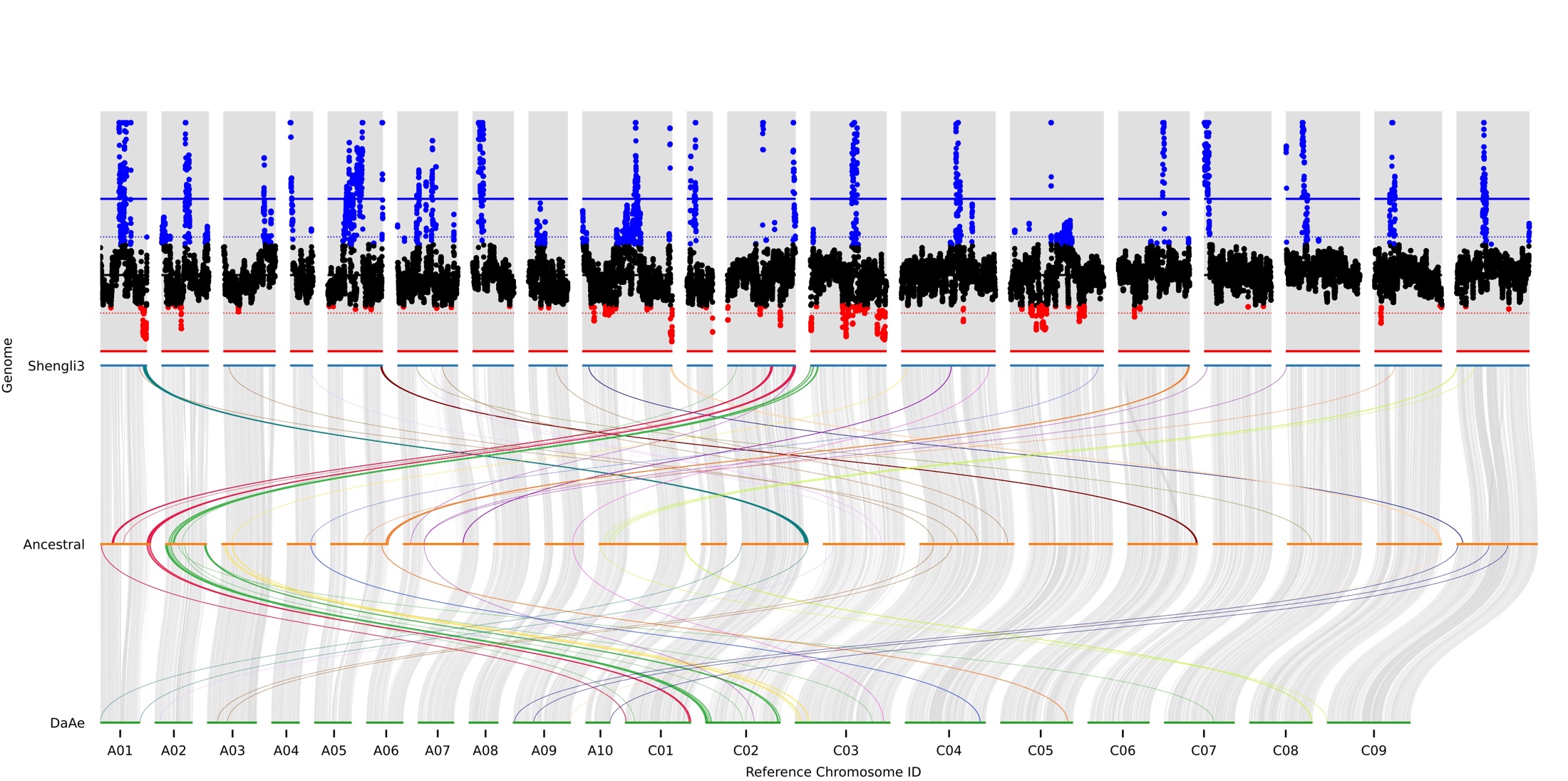


**Figure S26.** Coverage and homoeologous exchange plot. Top panel: Coverage of Da-Ae reads mapped to Shengli3; replotted from figures S3 – S21. Vertical lines indicate 0, 0.5x, 1x, 1.5x, and 2x coverage. Bottom panel: homoeologous exchange. Grey lines show homologous regions between the ancestral chromosomes and the two *B. napus* varieties. Colored lines indicate homoeologous exchange; the color of the line corresponds to the ancestral chromosome.


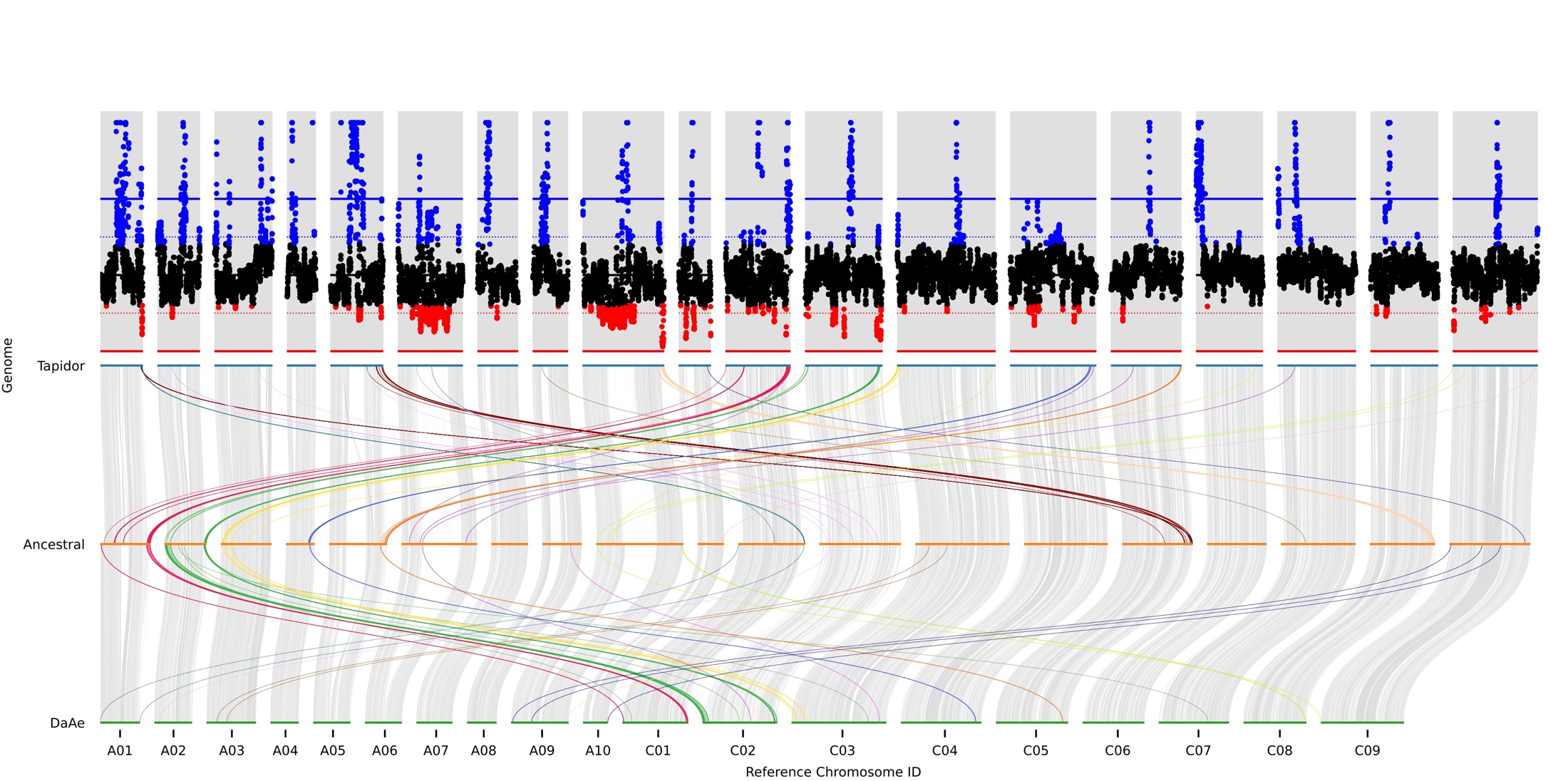


**Figure S27.** Coverage and homoeologous exchange plot. Top panel: Coverage of Da-Ae reads mapped to Tapidor; replotted from figures S3 – S21. Vertical lines indicate 0, 0.5x, 1x, 1.5x, and 2x coverage. Bottom panel: homoeologous exchange. Grey lines show homologous regions between the ancestral chromosomes and the two *B. napus* varieties. Colored lines indicate homoeologous exchange; the color of the line corresponds to the ancestral chromosome.


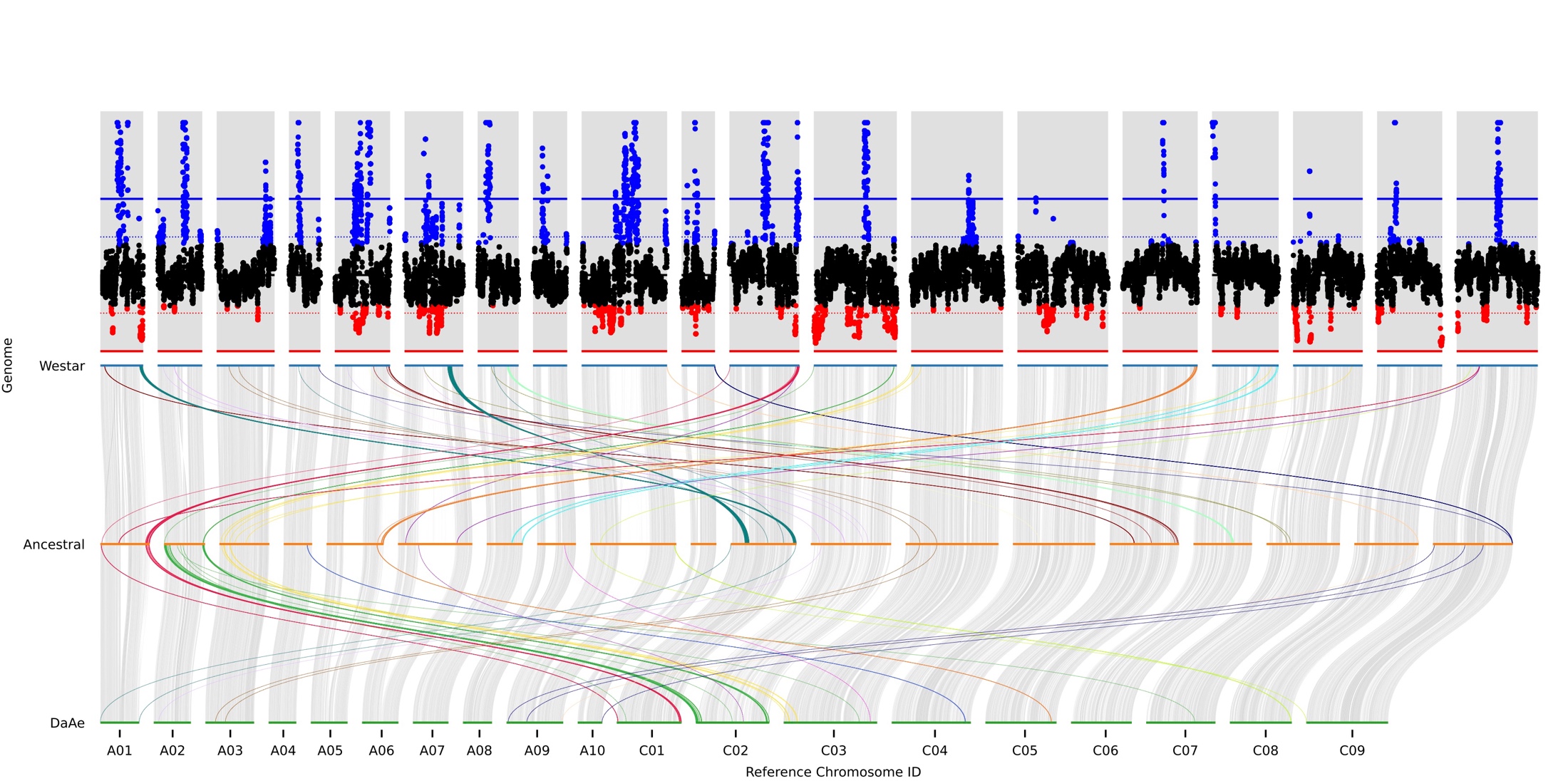


**Figure S28.** Coverage and homoeologous exchange plot. Top panel: Coverage of Da-Ae reads mapped to Westar; replotted from figures S3 – S21. Vertical lines indicate 0, 0.5x, 1x, 1.5x, and 2x coverage. Bottom panel: homoeologous exchange. Grey lines show homologous regions between the ancestral chromosomes and the two *B. napus* varieties. Colored lines indicate homoeologous exchange; the color of the line corresponds to the ancestral chromosome.


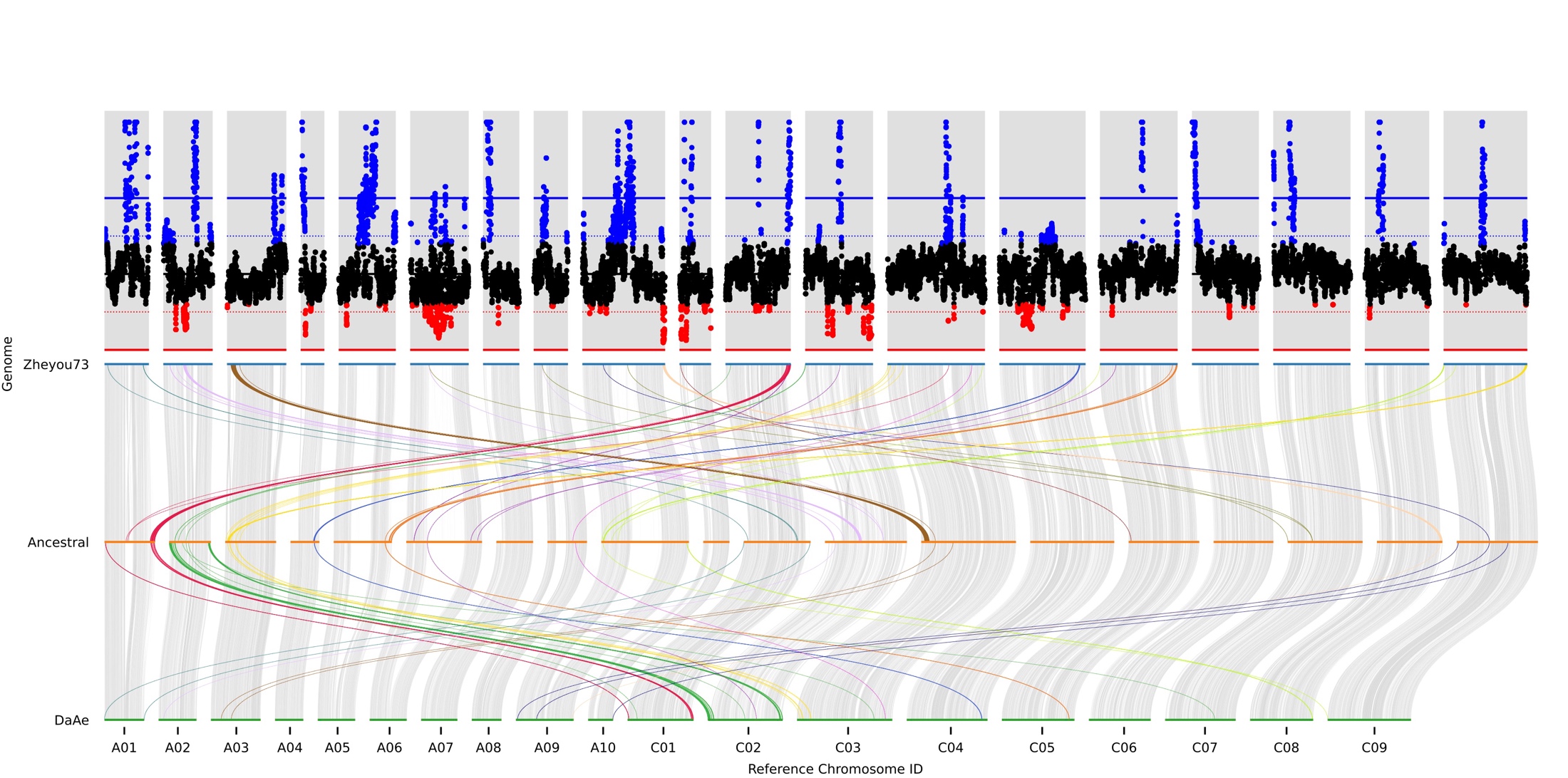


**Figure S29.** Coverage and homoeologous exchange plot. Top panel: Coverage of Da-Ae reads mapped to Zheyou73; replotted from figures S3 – S21. Vertical lines indicate 0, 0.5x, 1x, 1.5x, and 2x coverage. Bottom panel: homoeologous exchange. Grey lines show homologous regions between the ancestral chromosomes and the two *B. napus* varieties. Colored lines indicate homoeologous exchange; the color of the line corresponds to the ancestral chromosome.


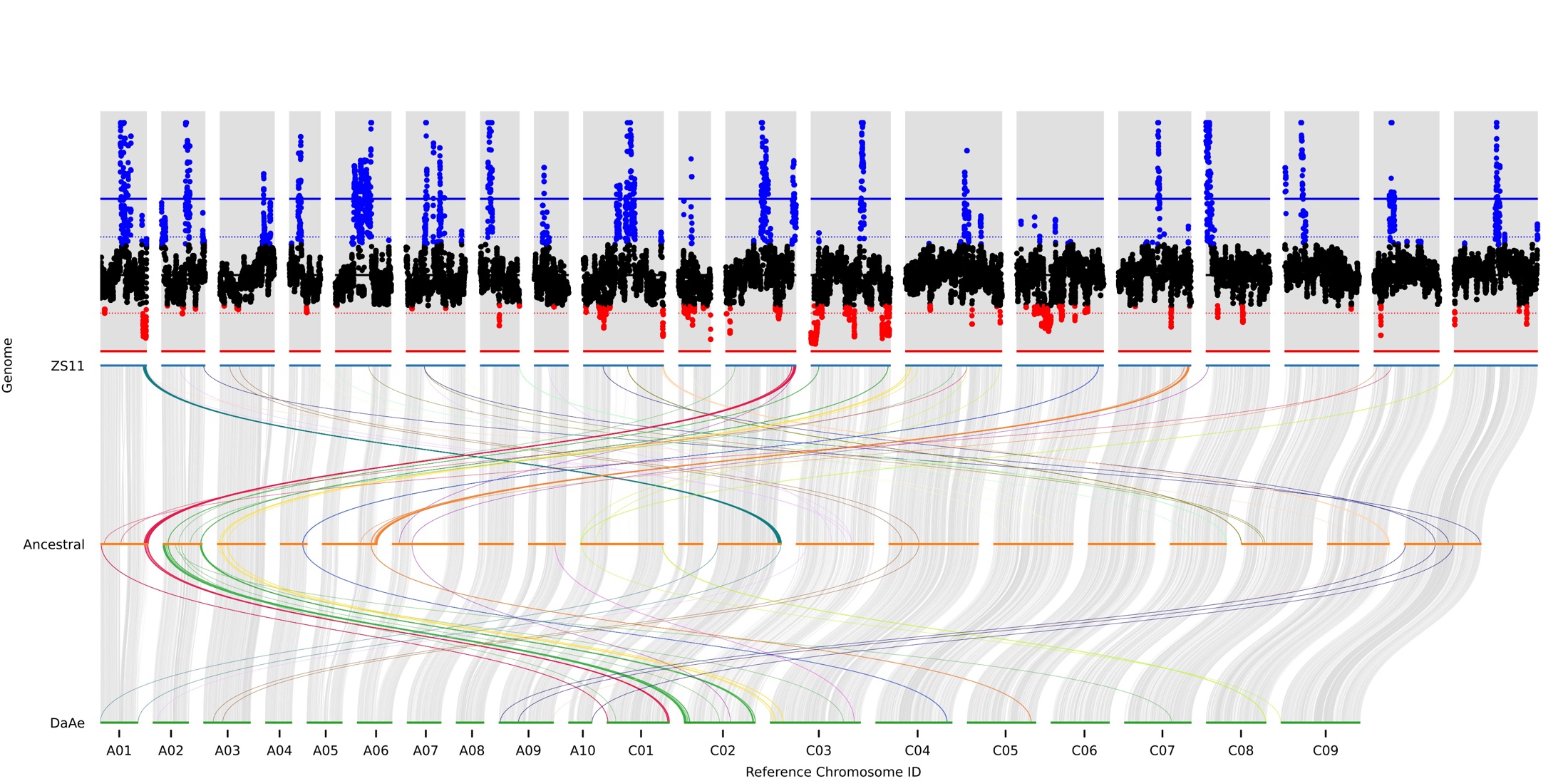


**Figure S30.** Coverage and homoeologous exchange plot. Top panel: Coverage of Da-Ae reads mapped to ZS11; replotted from figures S3 – S21. Vertical lines indicate 0, 0.5x, 1x, 1.5x, and 2x coverage. Bottom panel: homoeologous exchange. Grey lines show homologous regions between the ancestral chromosomes and the two *B. napus* varieties. Colored lines indicate homoeologous exchange; the color of the line corresponds to the ancestral chromosome.


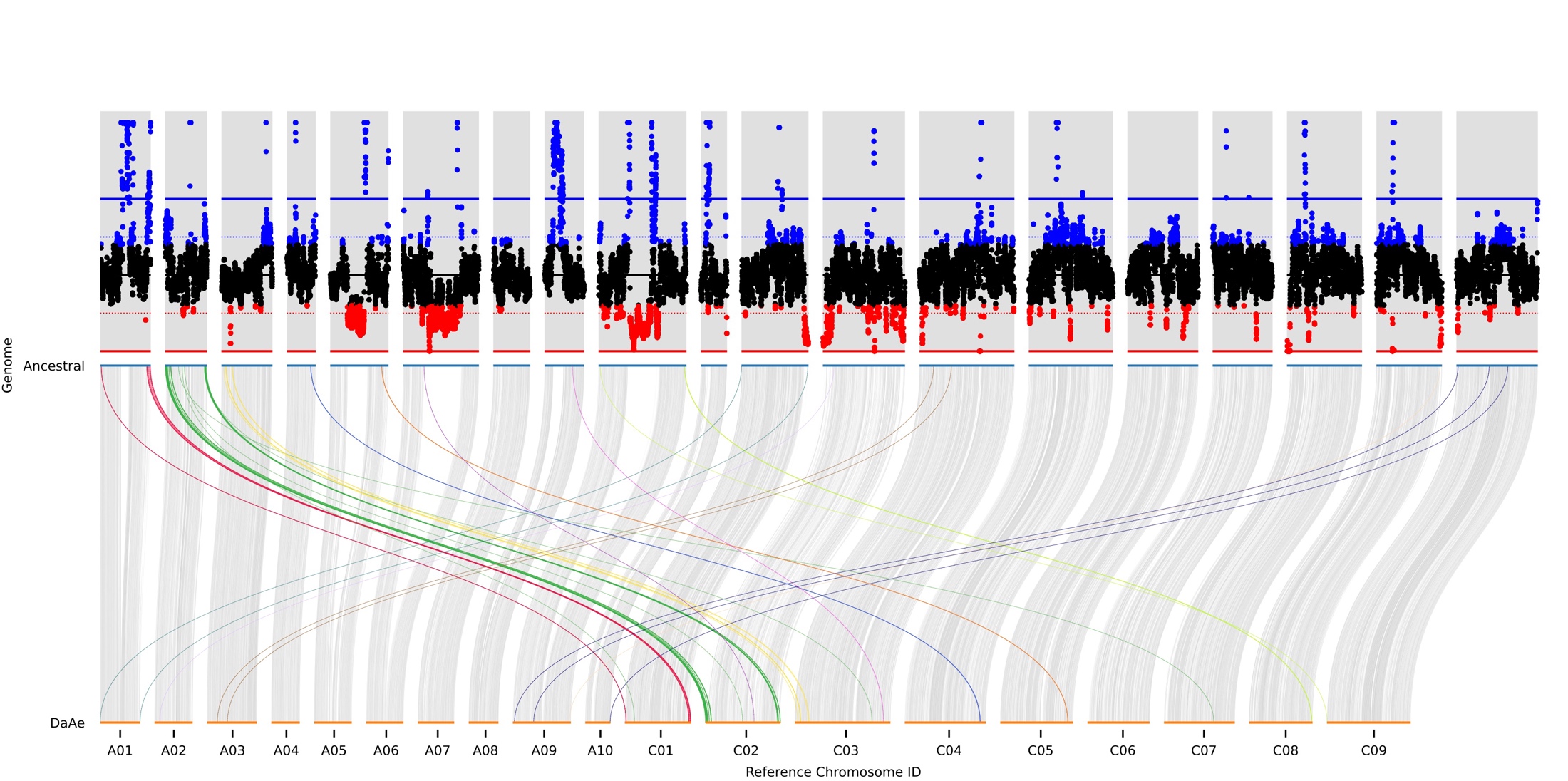


**Figure S31.** Coverage and homoeologous exchange plot. Top panel: Coverage of Da-Ae reads mapped to the “Ancestral” reference; replotted from figures S3 – S21. Vertical lines indicate 0, 0.5x, 1x, 1.5x, and 2x coverage. Bottom panel: homoeologous exchange. Grey lines show homologous regions between the ancestral chromosomes and Da-Ae. Colored lines indicate homoeologous exchange; the color of the line corresponds to the ancestral chromosome.


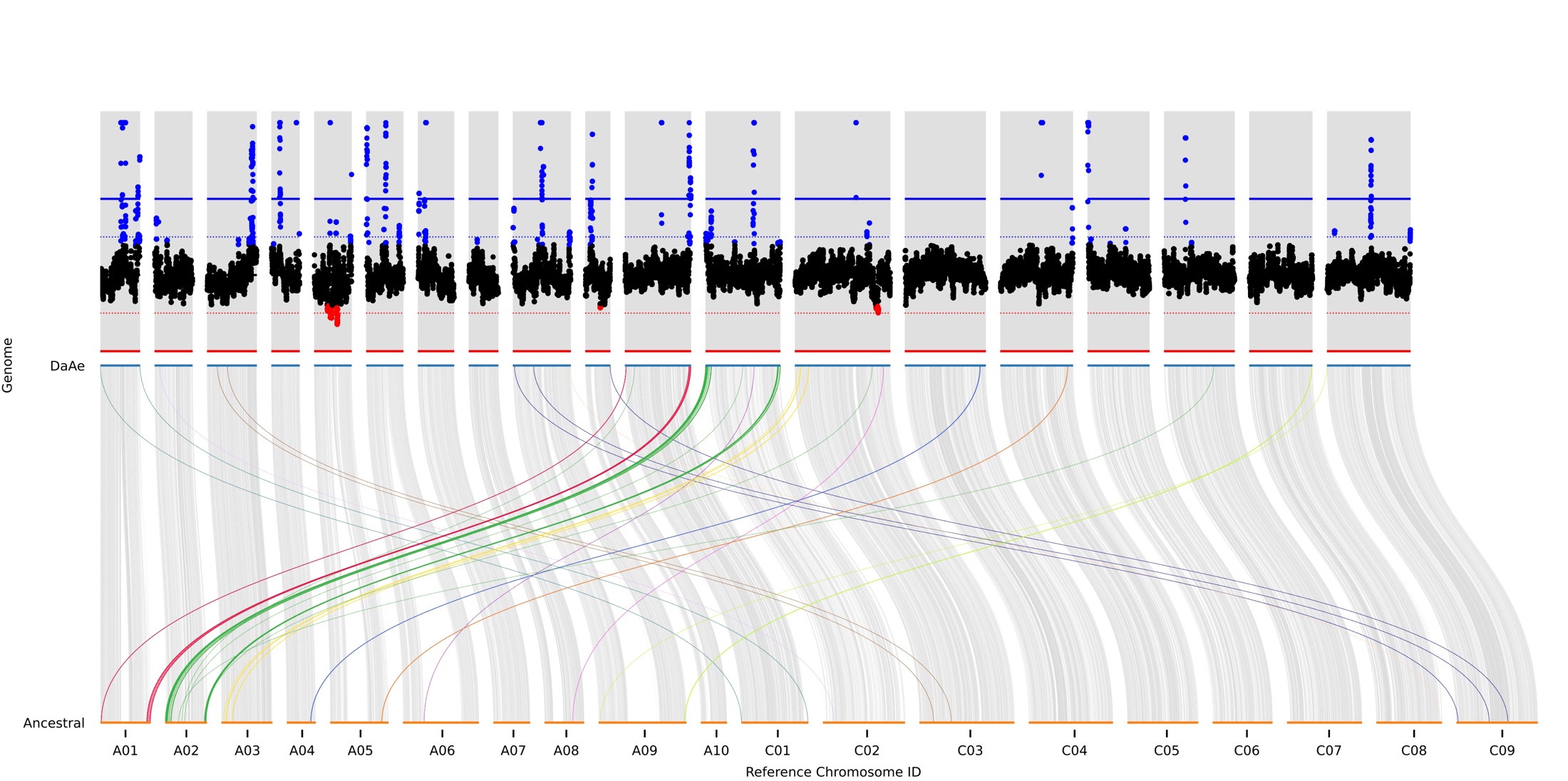


**Figure S32.** Coverage and homoeologous exchange plot. Top panel: Coverage of Da-Ae reads mapped to Da-Ae; replotted from figures S3 – S21. Vertical lines indicate 0, 0.5x, 1x, 1.5x, and 2x coverage. Bottom panel: homoeologous exchange. Grey lines show homologous regions between the ancestral chromosomes and Da-Ae. Colored lines indicate homoeologous exchange; the color of the line corresponds to the ancestral chromosome.

**Table S1.** Discrepancies between Da-Ae and Darmor-bzh assemblies. Discrepancy number, chromosome discrepancy is located on, type of discrepancy, data able to support Da-Ae’s composition, and action taken to resolve discrepancy.

| **Discrepancy** | **Chromosome** | **Type** | **Supported** | **Action taken** |
| --- | --- | --- | --- | --- |
| 1 | A01 | Inversion | Yes | None |
| 2 | A02 | Inversion | No | Flipped |
| 3 | A02 | Duplication | Yes | None |
| 4 | A03 | Inversion | No | Flipped |
| 5 | A04 | Inversion | Yes | None |
| 6 | A05 | Inversion | No | Flipped |
| 7 | A06 | Inversion | Yes | None |
| 8 | A07 | Inversion | Yes | None |
| 9 | A08 | Inversion | Yes | None |
| 10 | A09 | Gap | Yes | None |
| 11 | A10 | Inversion | Yes | None |
| 12 | C01 | Inversion | No | Flipped |
| 13 | C02 | Inversion | Yes | None |
| 14 | C03 | Inversion | No | Flipped |
| 15 | C04 | Inversion | Yes | None |
| 16 | C05 | Inversion | No | Flipped |
| 17 | C05 | Inversion | Yes | None |
| 18 | C06 | Inversion and Gap | No | Flipped and Joined |
| 19 | C06 | Inversion | No | Flipped |
| 20 | C06 | Inversion | Yes | None |
| 21 | C07 | Gap | No | Joined |
| 22 | C07 | Inversion | Yes | None |
| 23 | C08 | Duplication | Yes | None |
| 24 | C09 | Inversion | Yes | None |

**Table S2.** Transposable element content of three B. napus assemblies calculated using default parameters of repeatmasker v4.1.2-p1 and lib file bnapus.TE-families.fa located at (http://cbi.hzau.edu.cn/rape/download_ext/).

| **Comparison of the transposable elements among 3 B. napus genomes** |  |  |  |  |  |  |
| --- | --- | --- | --- | --- | --- | --- |
| **Classification** | **DaAe** | | **Darmor-Bzh_V10** | | **ZS11** | |
|  | Length (bp) | Percentage of genome (%) | Length (bp) | Percentage of genome (%) | Length (bp) | Percentage of genome (%) |
| **Class I: Retrotransposon** | 279891636 | 27.94725111 | 234603345 | 25.39558573 | 301651364 | 29.84025197 |
| SINE | 375932 | 0.037536906 | 67012 | 0.007253984 | 132092 | 0.013066934 |
| LINE | 29216324 | 2.917257389 | 27714254 | 3.000041255 | 28783458 | 2.847345452 |
| **LTR-Retrotransposon** | 250299380 | 24.99245681 | 206822079 | 22.38829049 | 272735814 | 26.97983958 |
| Copia | 82726692 | 8.260281256 | 65303382 | 7.069028092 | 82083435 | 8.119938032 |
| Gypsy | 104171553 | 10.40155609 | 92951519 | 10.06191225 | 103423820 | 10.23099252 |
| **Class II: DNA Transposon** | 41357988 | 4.12960563 | 38608863 | 4.179372167 | 41077363 | 4.063495175 |
| hAT | 2047996 | 0.204492922 | 1999669 | 0.21646224 | 2077757 | 0.205537915 |
| Harbinger | 4906031 | 0.489868444 | 4484716 | 0.48546618 | 4830866 | 0.477883663 |
| Unclassified | 218661515 | 21.83340794 | 204308974 | 22.1162493 | 219971502 | 21.76023658 |
| Total Content | 539911139 | 53.91026468 | 477521182 | 51.6912072 | 562700229 | 55.66398373 |

**Table S3.** Assembly statistics of intermediate assemblies along with BUSCOs percentages. BUSCOs percentages were calculated using the brassicales_odb10 dataset, which contains 4,596 BUSCOS

| **Assembly statistics of intermediate assemblies** |  |  |  |  |  |  |  |  |  |  |
| --- | --- | --- | --- | --- | --- | --- | --- | --- | --- | --- |
| **Assembly** | **N50 (Mbp)** | **Sequences** | **Total Length (Mbp)** | **Total Unambiguous Length (Mbp)** | **Complete BUSCOs** | **Complete single-copy BUSCOs** | **Complete duplicated BUSCOs** | **Fragmented BUSCOs** | **Missing BUSCOs** | **# BUSCOs** |
| DaAe Canu | 1.59 | 4,008 | 1,004 | 1,004 | 98.6 | 21.0 | 77.6 | 0.1 | 1.3 | 4596 |
| DaAe Pilon Canu | 1.59 | 4,008 | 1,004 | 1,004 | 98.6 | 18.7 | 79.9 | 0.1 | 1.3 | 4596 |
| DaAe Dovetail Pilon Canu | 42.79 | 3,190 | 1,004 | 1,004 | 98.6 | 19.9 | 78.7 | 0.1 | 1.3 | 4596 |
| DaAe Final | 48.21 | 3,164 | 1,002 | 1,001 | 98.5 | 18.0 | 80.5 | 0.2 | 1.3 | 4596 |

**Table S4.** Conserved homoeologous exchange genes. These genes were found in homoeologous exchange regions in all *B. napus* genome analyzed. The table also shows the best *Arabidopsis thaliana* hit (based on blastp) along with the annotation associated with that Arabidopsis gene.

**
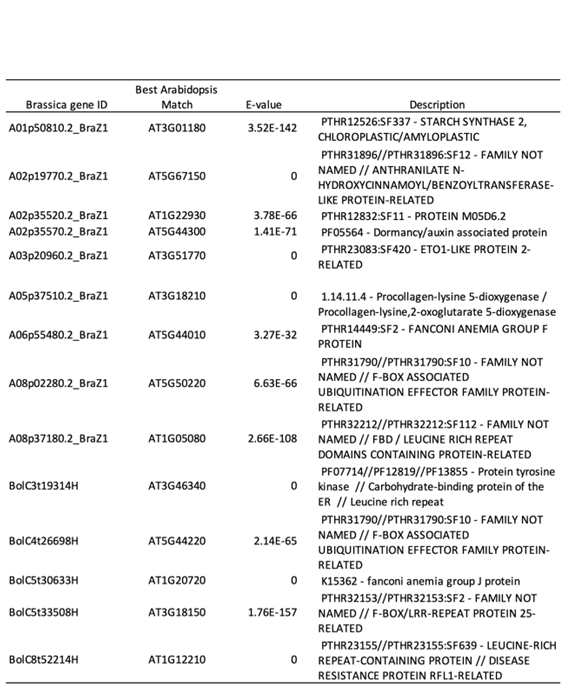
**
